# Photoinactivation of Catalase Sensitizes *Candida albicans* and *Candida auris* to ROS‐Producing Agents and Immune Cells

**DOI:** 10.1002/advs.202104384

**Published:** 2022-02-04

**Authors:** Pu‐Ting Dong, Yuewei Zhan, Sebastian Jusuf, Jie Hui, Zeina Dagher, Michael K. Mansour, Ji‐Xin Cheng

**Affiliations:** ^1^ Department of Biomedical Engineering Boston University Boston MA 02215 USA; ^2^ Photonics Center Boston University Boston MA 02215 USA; ^3^ Division of Infectious Diseases Massachusetts General Hospital Boston MA 02114 USA; ^4^ Harvard Medical School Boston MA 02115 USA

**Keywords:** *Candida auris*, photoinactivation of catalase, reactive oxygen species, synergistic therapy

## Abstract

Microbes have developed their own specific strategies to cope with reactive oxygen species (ROS). Catalase, a heme‐containing tetramer expressed in a broad range of aerobic fungi, shows remarkable efficiency in degrading hydrogen peroxide (H_2_O_2_) for fungal survival and host invasion. Here, it is demonstrated that catalase inactivation by blue light renders fungal cells highly susceptible to ROS attack. To confirm catalase as a major molecular target of blue light, wild type *Candida albicans* are systematically compared with a catalase‐deficient mutant strain regarding their susceptibility to ROS through 410 nm treatment. Upon testing a wide range of fungal species, it is found that intracellular catalase can be effectively and universally inactivated by 410 nm blue light. It is also found that photoinactivation of catalase in combination with ROS‐generating agents is highly effective in total eradication of various fungal species, including multiple *Candida auris *strains, the causative agent of the global fungal epidemic. In addition, photoinactivation of catalase is shown to facilitate macrophage killing of intracellular *Candida albicans*. The antifungal efficacy of catalase photoinactivation is further validated using a *C. albicans‐*induced mouse model of skin abrasion. Taken together, the findings offer a novel catalase‐photoinactivation approach to address multidrug‐resistant *Candida* infections.

## Graphical abstract

Most of the fungal microbes have evolved to scavenge H_2_O_2_ through expression of catalase. Here, it is found that catalase from most of pathogenic fungi (*Candida auris* included) can be inactivated by blue light, especially at 410 nm. Photoinactivation of catalase sensitizes these fungal cells highly susceptible to H_2_O_2_‐producing agents and immune cells.

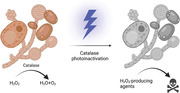

## 1 Introduction

The frequency of invasive fungal infections in immunocompromised patients has been consistently increasing over the past few decades.^[^ ^1^ ^]^ *Candida* species are the most common cause of human fungal infections,^[^ ^2^ ^]^ such as oropharyngeal, cutaneous candidiasis, and mucosal or deep‐seated organ infections.^[^ ^3^ ^]^ Invasive *Candida* infections remain a significant cause of morbidity and mortality among immunocompromised patients, partly due to the growing spread of antifungal resistance.^[^ ^4^ ^]^ The growing emergence of multidrug‐resistant *Candida* species poses an alarming trend worldwide.^[^ ^5^ ^]^ Especially of concern is *Candida auris* (*C. auris*), which has resulted in multiple worldwide healthcare outbreaks with record‐breaking mortality rate due to its multi‐drug or even pan‐drug resistance nature and biofilm formation.^[^ ^6^ ^]^ Ongoing COVID‐19 pandemic has further accelerated *C. auris* outbreaks.^[^ ^7^ ^]^ Life‐threatening invasive mycosis has been recorded to accompany COVID‐19 patients due to impaired immune responses, forcing higher than normal usage of antifungal agents.^[^ ^8^ ^]^ At a higher level, the long‐term imprudent usage of antifungal agents has been shown to accelerate the resistance of antifungal development.^[^ ^9^ ^]^ Confronted with this severe situation, novel therapeutic approaches are highly desired.

Antimicrobial blue light, especially in the 400–430 nm range, has drawn increasing attention in recent years as a nondrug approach to treat wide‐ranging bacterial^[^ ^10^ ^]^ and fungal infections.^[^ ^11^ ^]^ The antifungal efficacy of blue light has been studied by many research groups worldwide. Blue light at 415 nm has demonstrated the capability to inactivate *Candida albicans* both in vitro and in vivo mouse burn infection models, and the susceptibility of *C. albicans* to blue light‐induced inactivation did not change even after the 10^th^ passage in the presence of blue light exposure suggesting the unlikelihood of fungi developing photoresistance.^[^ ^12^ ^]^ Gupta et al. achieved 4.52‐log_10_ reduction of *C. albicans* after delivering 332.1 J cm^−2^ of 405 nm irradiance.^[^ ^13^ ^]^ Rosa et al. reported a 2.3‐log_10_ reduction of *C. albicans* in a biofilm setting under the treatment of 455 nm exposure with a dose of 45.16 J cm^−2^.^[^ ^14^ ^]^ Ferrer‐Espada et al. further demonstrated significant antimicrobial effect of 405 nm on *C. albicans* both in monomicrobial and polymicrobial biofilm schemes.^[^ ^15^ ^]^ Blue light was also utilized to combine with other agents to eliminate *C. albicans*. Leanse et al. showed that quinine chloride could enhance the fungicidal effects of blue light 405 nm on the elimination of *C. albicans* both in vitro and in vivo. Besides *C. albicans*, blue light can also inhibit the growth of microconidia of molds, such as *Trichophyton rubrum* and *Trichophyton mentagrophytes*.^[^ ^16^ ^]^ Studies also demonstrated that 405 nm light has detrimental effect on additional yeasts such as *Sacchromyces cerevisiae* and obligate molds including *Aspergillus niger*.^[^ ^17^ ^]^ Noteworthy, no significant loss of viability on human keratinocytes was observed after 216 J cm^−2^ blue light treatment.^[^ ^15^ ^]^ Collectively, these studies demonstrate that blue light works effectively against major pathogenic fungal species without developing resistance and no significant cytotoxicity on host cells, thus emerging as a drug‐free alternative approach to combat fungal infections.

However, the underlying antimicrobial mechanisms of blue light has stayed elusive for decades. A widely believed hypothesis is that metal‐free porphyrin (in the 405–420 nm window) or riboflavin (in the 450–470 nm window) are endogenous molecular targets.^[^ ^11a^ ^]^ Bacterial/fungal inactivation are eradicated by the reactive oxygen species (ROS) produced through photodynamic reaction between these molecules and blue light.^[^ ^18^ ^]^ However, this perspective remains controversial. First, it has been reported that the total amount of protoporphyrins inside the microbes was not a contributing factor to the antimicrobial efficacy of blue light.^[^ ^19^ ^]^ Second, the intracellular concentration of porphyrins or riboflavin is as low as 2–4 × 10^−3^ mg mL^−1^.^[^ ^20^ ^]^ Given the situation that research of antimicrobial blue light on fungi stays understudied and the working mechanism of antimicrobial blue light has yet to be clarified, other possible molecular targets are assumed to exist.

Here, using the wild type *C. albicans* SC5314 along with a catalase‐deficient *C. albicans* mutant, we demonstrate that catalase is a major molecular target of antimicrobial blue light (400–420 nm window). Catalase from wide‐ranging fungal species could be effectively inactivated by 410 nm blue light. Subsequently, photoinactivation of catalase renders fungal cells highly susceptible to nonfungicidal low‐concentration hydrogen peroxide (H_2_O_2_) and ROS‐producing antifungal drugs. Moreover, the synergy between photoinactivation of catalase and H_2_O_2_ enables total eradication of multiple notorious drug‐resistant *C. auris* isolates whereas either alone has limited efficacy. Furthermore, photoinactivation of catalase substantially boosts macrophages activity against *C. albicans* indicated by shorter hyphae length and higher eradication percent of *C. albicans* were observed in the case of 410 nm involved group. Finally, we validated the synergistic effect between photoinactivation of catalase and H_2_O_2_ in a *C. albicans*‐induced mouse skin abrasion model without noticeable skin damage. Collectively, our findings provide a novel catalase‐targeting strategy to treat multidrug resistant fungal infections.

## 2 Results

### 2.1 Catalase Inside Fungal Cells Can Be Inactivated by Blue Light Irradiance

**Figure 1:**
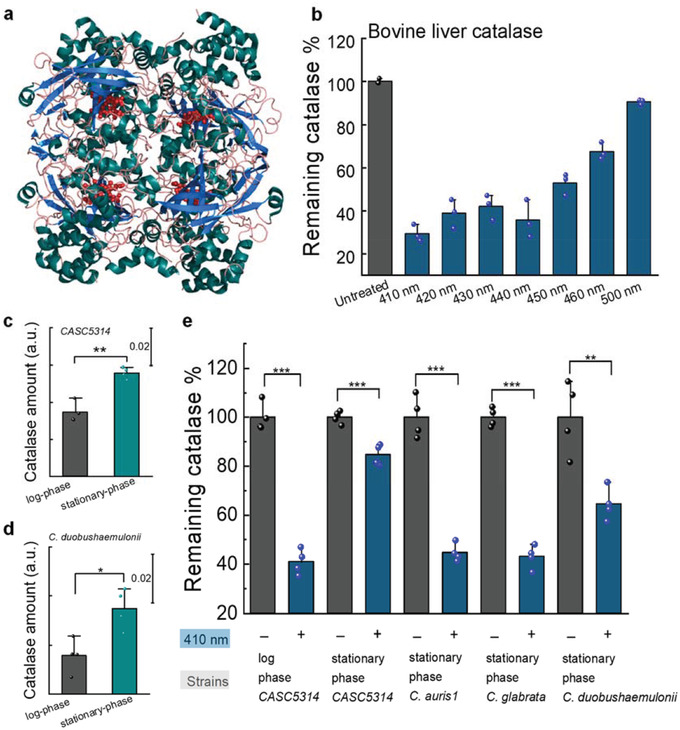
Catalase from bovine liver and fungal cells can be inactivated by blue light. a) Structure of bovine liver catalase revealed by PyMOL simulation. PDB ID: 1TGU. b) Quantification of remaining catalase percentage of bovine liver catalase (2.5 U mL^−1^) by Amplex red catalase assay under different treatment schemes. *N* = 3. Dose: 15 J cm^−2^. c,d) Comparison of catalase amount between log‐ and stationary‐ phase *C. albicans* SC5314 (c) and *Candida duobushaemulonii* (d). e) Quantification of remaining catalase percentage in various fungal strains with and without 410 nm blue light. Dose: 30 J cm^−2^. Data: Mean + SD from at least three replicates. Student unpaired *t*‐test. *: *p* < 0.05, **: *p* < 0.01; ***: *p* < 0.001.

As shown in **Figure** **1**a, catalase (from bovine liver) revealed by PyMOL has a tetramer structure, with porphyrin rings hiding inside its hydrophobic pocket. It was reported as early as in 1965 that bovine liver catalase can be inactivated by visible light exposure.^[^ ^21^ ^]^ Nonetheless, photoinactivation of intracellular catalase remained underexplored. To better understand how photons inactivate catalase, especially catalase inside fungal cells, we first quantified the remaining catalase percentage from bovine liver catalase (2.5 U mL^−1^) after blue light exposure under various wavelengths using an Amplex red catalase kit. And we found that blue light, especially in the 400–420 nm window, inactivates catalase by 70% compared to the untreated group (Figure 1b). 410 nm shows the best capability to inactivate catalase, which might be due to the fact that the optimal absorption peak of catalase is around 410 nm. In the following experiments, we used continuous wave (CW) 410 nm LED as the light source to inactivate catalase.

To query whether fungal catalase can be inactivated by blue light in the same way as liver catalase, we tested the remaining catalase percentage inside the fungal cells after 410 nm exposure. Prior to that, we investigated the catalase expression level for fungal cells at different metabolic phases. It was reported that stationary‐phase microbes usually have higher amount of sigma factor *σ* ^S^, the key protein responsible for survival and improved resistance under stressful conditions.^[^ ^22^ ^]^ Increased expression of *σ* ^*S*^ leads to the relatively higher amount of catalase in the stationary‐phase microbes.^[^ ^22^ ^]^ As shown in Figure 1c,d, we indeed found that stationary‐phase *Candida spp*. have more catalase compared to log‐phase. Of note, catalase in log‐ and stationary‐phase *C. albicans* could be consistently inactivated by 410 nm exposure. Moreover, this inactivation behavior exists in many other fungal strains apart from *C. albicans* (Figure 1e). In short, we found that catalase inside fungal cells can be effectively inactivated by 410 nm exposure regardless of metabolic phases.

### 2.2 Photoinactivation of Catalase Sensitizes *C. albicans* to H_2_O_2_

**Figure 2:**
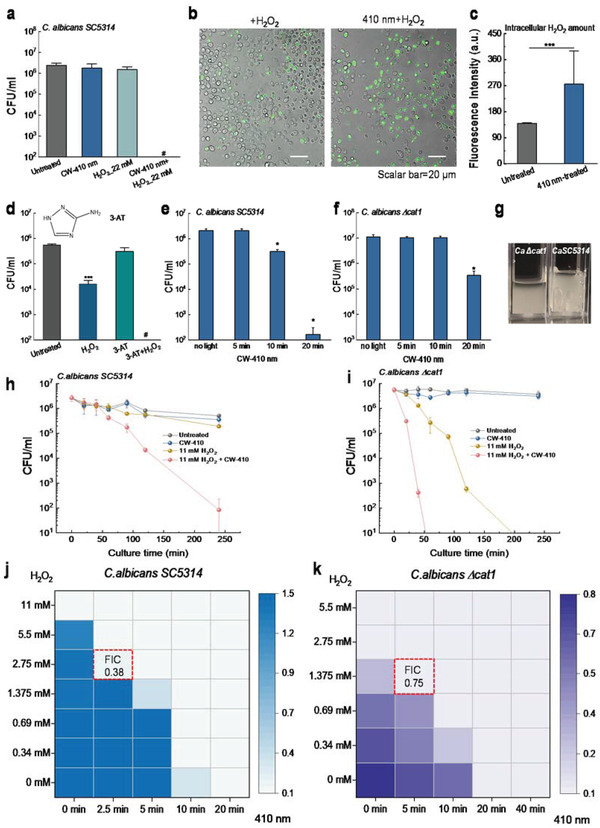
Photoinactivation of catalase sensitizes *C. albicans* to H_2_O_2_ and catalase is identified as a primary molecular target of blue light. a) Colony‐forming unit (CFU/mL) of *C. albicans* SC5314 under different treatment schemes. b) Confocal imaging of intracellular H_2_O_2_ with/without 410 nm exposure. Scalar bar = 20 µm. H_2_O_2_: 22 × 10^−3^ m, images were taken after 30 min incubation and washing step. c) Quantitation of intracellular H_2_O_2_ via fluorescence intensity from images acquired in (b). d) CFU/mL of *C. albicans* SC5314 in the presence of 3‐AT. Significant difference from the untreated group. 3‐AT: 50 × 10^−3^ m, 4 h incubation. e,f) CFU/Ml of *C. albicans* SC5314 and its catalase‐deficient mutant *C. albicans* Δ*cat1* under 410 nm exposure with various doses. g) Pictures showing bubble formation phenomena of *C. albicans* Δ*cat1* and *C. albicans SC5314* in the presence of 3% H_2_O_2_. H,i) Time‐killing curves of *C. albicans* SC5314 (h) and *C. albicans* Δ*cat1* (i) under different treatment schemes. j,k) Checkerboard broth dilution assay showing the combinatorial behavior between blue light and H_2_O_2_ against *C. albicans* SC5314 and *C. albicans* Δ*cat1*. Data: Mean + SD. *N* = 3. Student unpaired *t*‐test. *: *p* < 0.05, **: *p* < 0.01; ***: *p* < 0.001. Pound sign (#) means CFU results are below the detection limit.

Having established that fungal catalase can be efficiently inactivated by 410 nm blue light, we then queried whether this attenuation effect sensitizes *C. albicans* to H_2_O_2_ considering the major function of catalase. A colony‐forming unit (CFU/mL) assay was conducted by combining 410 nm with H_2_O_2_ at a nonfungicidal concentration (22 × 10^−3^ m). As shown in **Figure** **2**a, photoinactivation of catalase significantly sensitizes wild type *C. albicans* SC5314 to H_2_O_2_ by around six orders of magnitude, whereas H_2_O_2_ or blue light alone did not have apparent killing effect. This result indicates a synergistic killing effect between photoinactivation of catalase and H_2_O_2_. To understand whether this augmented killing was due to intracellular accumulation of H_2_O_2_ after catalase inactivation, we utilized an established kit using H_2_O_2_‐sensitive fluorophore to quantify intracellular H_2_O_2_ in *C. albicans* cells treated with H_2_O_2_ or 410 nm plus H_2_O_2_ through a confocal laser scanning microscope (CLSM). As shown in Figure 2b,c, fluorescent intensity in the 410 nm plus H_2_O_2_ treated single *C. albicans* SC5314 was significantly higher than cells treated by H_2_O_2_ alone, suggesting that photoinactivation of catalase boosts intracellular H_2_O_2_ accumulation.

To investigate whether catalase is the primary target for blue light exposure, we conducted the following experiments. First, a catalase inhibitor, 3‐Amino‐1,2,4‐triazole (3‐AT),^[^ ^23^ ^]^ was utilized to chemically inhibit catalase activity within *C. albicans*, and then the susceptibility to H_2_O_2_ was recorded. As shown in Figure 2d, total eradication was obtained when adding H_2_O_2_ to 3‐AT treated *C. albicans* SC5314 whereas 3‐AT alone only exerts limited killing efficiency, suggesting the pivotal role that catalase plays in neutralizing H_2_O_2_. Second, we exploited a catalase‐deficient *C. albicans* strain, *C. albicans* Δ*cat1*, to further evaluate the function of catalase underlying the antimicrobial effect of blue light. *C. albicans* Δ*cat1* demonstrates significantly lower susceptibility to 410 nm blue light compared to its the wild type (Figure 2e,f), indicating catalase‐mediated sensitivity to 410 nm blue light.

Next, we examined the time‐kill CFU assay of wild type *C. albicans* SC5314 and catalase‐deficient *C. albicans* Δ*cat1* under different treatment schemes. *C. albicans* Δ*cat 1* did not produce the typical oxygen bubbles as the wild type *C. albicans* SC5314 did in the presence of H_2_O_2_ (Figure 2g), confirming the major role of catalase in H_2_O_2_ neutralization. As shown in Figure 2h, 4 h after treatments, both H_2_O_2_ and 410 nm blue light alone did not exert significant fungicidal effects, whereas H_2_O_2_ plus 410 nm blue light‐treated group had around a 5‐log_10_ reduction of wild type *C. albicans* SC5314. These data suggest that there is an ample synergy between photoinactivation of catalase and H_2_O_2_ against *C. albicans*. Of note, when applying H_2_O_2_ with the same concentration used to treat wild type *C. albicans* SC5314, H_2_O_2_ alone achieved more than 5‐log_10_ reduction of *C. albicans* Δ*cat1* (Figure 2i; Figure S1a, Supporting Information). Moreover, *C. albicans* Δ*cat1* exhibited similar susceptibility to H_2_O_2_ killing compared to wild type *C. albicans* SC5314 exposed to 410 nm plus H_2_O_2_ in a time‐kill assay, corroborating that catalase is a key target of 410 nm light. Noteworthy, enhanced H_2_O_2_ killing of *C. albicans* Δ*cat1* after 410 nm exposure was observed (Figure 2i; Figure S1b), hinting that in the absence of catalase, cells could express other molecular targets that are responsive to 410 nm light exposure.

To further confirm the synergistic effect between photoinactivation of catalase and H_2_O_2_, we conducted a checkerboard broth dilution assay to derive a fractional inhibitory concentration index (FICI).^[^ ^24^ ^]^ FICI was calculated through the following equation

(1) $$\mathrm{FICI}\;=\;\mathrm{FI}C_{A}+\;\mathrm{FI}C_{B}=\frac{{\mathrm{MIC}}_{A}^{\mathrm{Comb}}}{{\mathrm{MIC}}_{A}^{\mathrm{alone}}}\;+\frac{{\mathrm{MIC}}_{B}^{\mathrm{Comb}}}{{\mathrm{MIC}}_{B}^{\mathrm{alone}}}$$

where ${\mathrm{MIC}}_{A}^{\mathrm{alone}}$ and ${\mathrm{MIC}}_{B}^{\mathrm{alone}}$ are the minimal inhibitory concentrations (MICs) of the drugs A and B when functioning alone, and ${\mathrm{MIC}}_{A}^{\mathrm{Comb}}$ and ${\mathrm{MIC}}_{B}^{\mathrm{Comb}}$ are the MICs of drugs A and B in combination, respectively. A synergy between two agents is defined as FICI ≤ 0.5,^[^ ^25^ ^]^ and “no interaction” as an FICI in the range of 0.5–4.^[^ ^25^ ^]^ The MICs of 410 nm against *C. albicans* SC5314 and *C. albicans* Δ*cat1* were 282 and 564 J cm^−2^, respectively (Figure S2a,c, Supporting Information). And MICs of H_2_O_2_ were 11 and 2.75 × 10^−3^ m, respectively (Figure S2b,d, Supporting Information). Once combining 410 nm and H_2_O_2_ together, MIC of H_2_O_2_ against wild type *C. albicans* SC5314 was around 2.75 × 10^−3^ m (Figure 2j), and 1.375 × 10^−3^ m against *C. albicans* Δ*cat 1* (Figure 2k). FICI from both strains could be calculated from the above equation. Then we obtained an FICI of 0.38 in the case of wild type *C. albicans* SC5314, and an FICI of 0.75 for *C. albicans* Δ*cat1*. These results indicate a strong synergy between photoinactivation of catalase and H_2_O_2_ in eradicating *C. albicans*.

### 2.3 Photoinactivation of Catalase Enhances ROS‐Generating Agents to Inhibit the Proliferation of Wide‐Ranging Fungal Cells

After demonstrating the synergy between photoinactivation of catalase with nonfungicidal low‐concentration H_2_O_2_ against *C. albicans*, we next query whether this synergy still holds effective against other fungal species. To have a high‐throughput analysis, we adopted a PrestoBlue viability assay^[^ ^26^ ^]^ to investigate the time‐course proliferation of wide‐ranging fungal species under different treatment schemes (Figures S3 and S4, Supporting Information).

**Figure 3:**
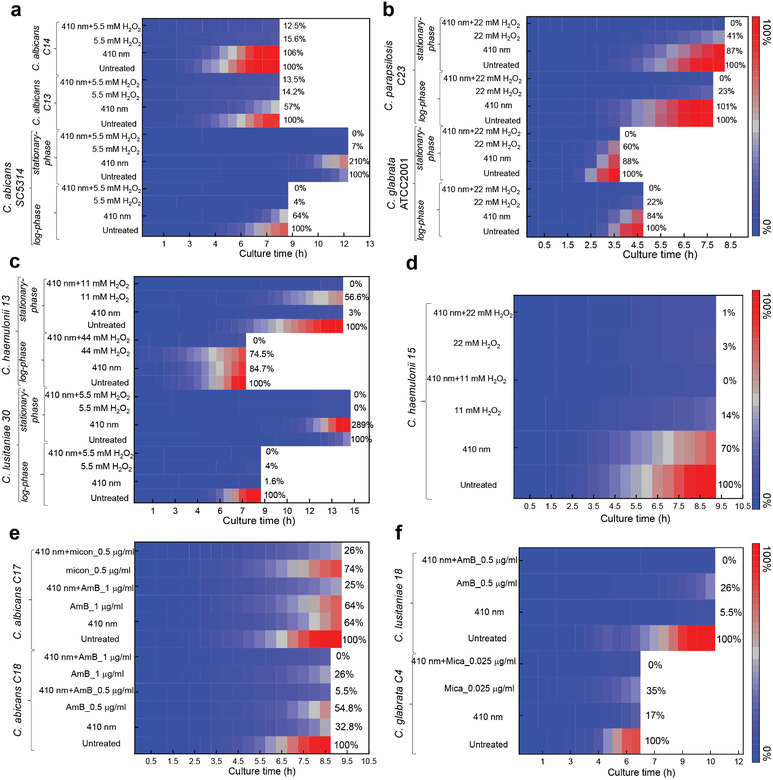
Photoinactivation of catalase enhances ROS‐generating agents to inhibit the proliferation of wide‐ranging fungal cells. Data were acquired through PrestoBlue proliferation assay and were presented as mean from three replicates. Blue light: 410 nm, 30 J cm^−2^. Abbreviations: AmB (amphotericin B), micon (miconazole), mica (micafungin).

As shown in **Figure** **3**a, photoinactivation of catalase consistently enhances the fungistatic effect of H_2_O_2_ against both log‐ and stationary‐phase *C. albicans* SC5314, and *C. albicans* C13, *C. albicans* C14 as indicated by nearly 0% proliferation rate in the combination‐treated group. Besides *C. albicans*, we also investigated whether this enhancement works for non‐albicans *Candida* species. *Candida glabrata* (*C. glabrata*), the commensal saprophyte, caused increasing incidence and prevalence of mucosal and systemic infections due to the widespread use of immunosuppressive and antimycotic therapy.^[^ ^27^ ^]^ *Candida parapsilosis* (*C. parapsilosis*), the second most commonly isolated *Candida* species from blood cultures, could also cause invasive *C. parapsilosis* infections in both AIDS patients and surgical patients.^[^ ^28^ ^]^ *Candida lusitaniae* (*C. lusitaniae*), fungal pathogen capable of developing both acute and long‐term infections, can rapidly develop resistance ^[^ ^29^ ^]^ to multiple antifungals and lead to the breakouts of multidrug‐resistant fungal infections.^[^ ^30^ ^]^ *Candida haemulonii* (*C. haemulonii*), the opportunistic fungal pathogen associated with bloodstream infections, has emerged as a multi‐drug resistant yeast for deep‐seated soft tissue and bone infections in diabetic patients.^[^ ^31^ ^]^ Thus, we further wondered whether photoinactivation of catalase enhances the antifungal efficacy of H_2_O_2_ against these fungal species.

Noteworthy, complete inhibition of proliferation (0% growth compared to the untreated group) was obtained from all the above‐mentioned fungal species in the 410 nm plus H_2_O_2_ treated group (Figure 3b–d; Figure S3, Supporting Information), both in the form of log‐ and stationary‐phase. For example, in the case of stationary‐phase *C. glabrata* ATCC2001 (Figure 3b), 410 nm exposure (30 J cm^−2^) inhibited around 12% of the original fungal cells to proliferate. At a concentration of 22 × 10^−3^ m, H_2_O_2_ alone prohibited the growth of around 40% of the original fungal cells. Once combining these two treatments together, total growth inhibition was obtained. These findings consolidated the synergy between photoinactivation of catalase and H_2_O_2_ against those catalase‐positive fungal pathogens, which holds clinical potential for treating multidrug‐resistant fungal infections.

To treat fungal infections in the clinic, currently there are limited classes of antifungals available, such as liposomal formulations of amphotericin B (representative of polyenes), miconazole (azole representative), and echinocandin (antifungal which inhibits the cell wall synthesis). Interestingly, it was reported that all these antifungals are able to induce an intracellular ROS burst within fungal cells as part of their proposed antifungal mechanism.^[^ ^32^ ^]^ Considering the pivotal role of catalase in scavenging ROS^[^ ^33^ ^]^ and the fact that fluconazole and amphotericin‐B resistance are associated with increased expression of catalase and superoxide dismutase,^[^ ^34^ ^]^ we reasoned that photoinactivation of catalase could enhance the antifungal efficacy of these antimycotics against fungal cells. The same time‐course PrestoBlue proliferation assay was applied for the evaluation when treating fungal cells with these antifungals at a sublethal concentration (Figure S4, Supporting Information).

As shown in Figure 3e, in the case of *C. albicans* C17, 410 nm blue light alone (30 J cm^−2^) inhibited around 36% of original *C. albicans* to divide, amphotericin B (AmB, 1 µg mL^−1^) alone also gave out around 36% inhibition effects, whereas the administration of AmB after 410 nm blue light treatment drastically suppressed fungal growth by 75%. Similar phenomenon was found in the combinational behavior between 410 nm treatment and miconazole (micon, 0.5 µg mL^−1^) against *C. albicans* (Figure 3e). Micafungin (mica, 0.025 µg mL^−1^) has demonstrated the augmented inhibition effect induced by photoinactivation of catalase against *C. glabrata* as well (Figure 3f). In combination with the time‐course calibration curves with known number of fungal cells under the same conditions, we can derive the relatively proliferated fungal cell numbers (Figures S5 and S6, Supporting Information). Through comparison between the proliferated fungal cell numbers under different treatment schemes, we can clearly observe the enhanced inhibition effect by 410 nm (Figure S6, Supporting Information). Collectively, these data were in line with our hypothesis, that is, photoinactivation of catalase boosts the antimycotic efficacy of commonly used antifungal agents.

### 2.4 Elimination of *C. auris* by Synergizing Photoinactivation of Catalase with H_2_O_2_

*C. auris*, as an emerging and particularly notable *Candida* species, has been associated with nosocomial outbreaks on five continents since 2009 due to its innate resistance to multiple classes of antifungal drugs.^[^ ^35^ ^]^ Its high morbidity and mortality have led to it being one of the major public health concerns worldwide. *C. auris* demonstrates a unique propensity to colonize and persist on various surfaces, contributing to multiple outbreaks in health care settings.^[^ ^36^ ^]^ *C. auris* has also been reported to cause bloodstream infections and urinary tract infections for patients with COVID‐19,^[^ ^7^, ^36^ ^]^ thus likely as a compounding factor in COVID‐19 pandemic. Most of the clinical isolated *C. auris* strains exhibit resistance to triazoles (fluconazole) and other antifungal drugs.^[^ ^37^ ^]^ Confronted with this situation, it is imperative to have alternative approaches to both sterilize clinical settings and further treat *C. auris*‐caused infections.

*C. auris* strains have been reported to be extremely resistant to hydrogen peroxide vapor sterilization,^[^ ^38^ ^]^ this triggered us to wonder whether this persistence is due to catalase. To investigate whether photoinactivation of catalase and H_2_O_2_ could efficiently kill *C. auris*, we conducted CFU assay of multiple *C. auris* clinical isolates acquired from antimicrobial resistance bank (AR‐BANK) of the Centers for Disease Control and Prevention. Here *C. auris* 1, 2, 5, 6 represent AR‐BANK#0381, 0382, 0385, 0386, respectively.

**Figure 4:**
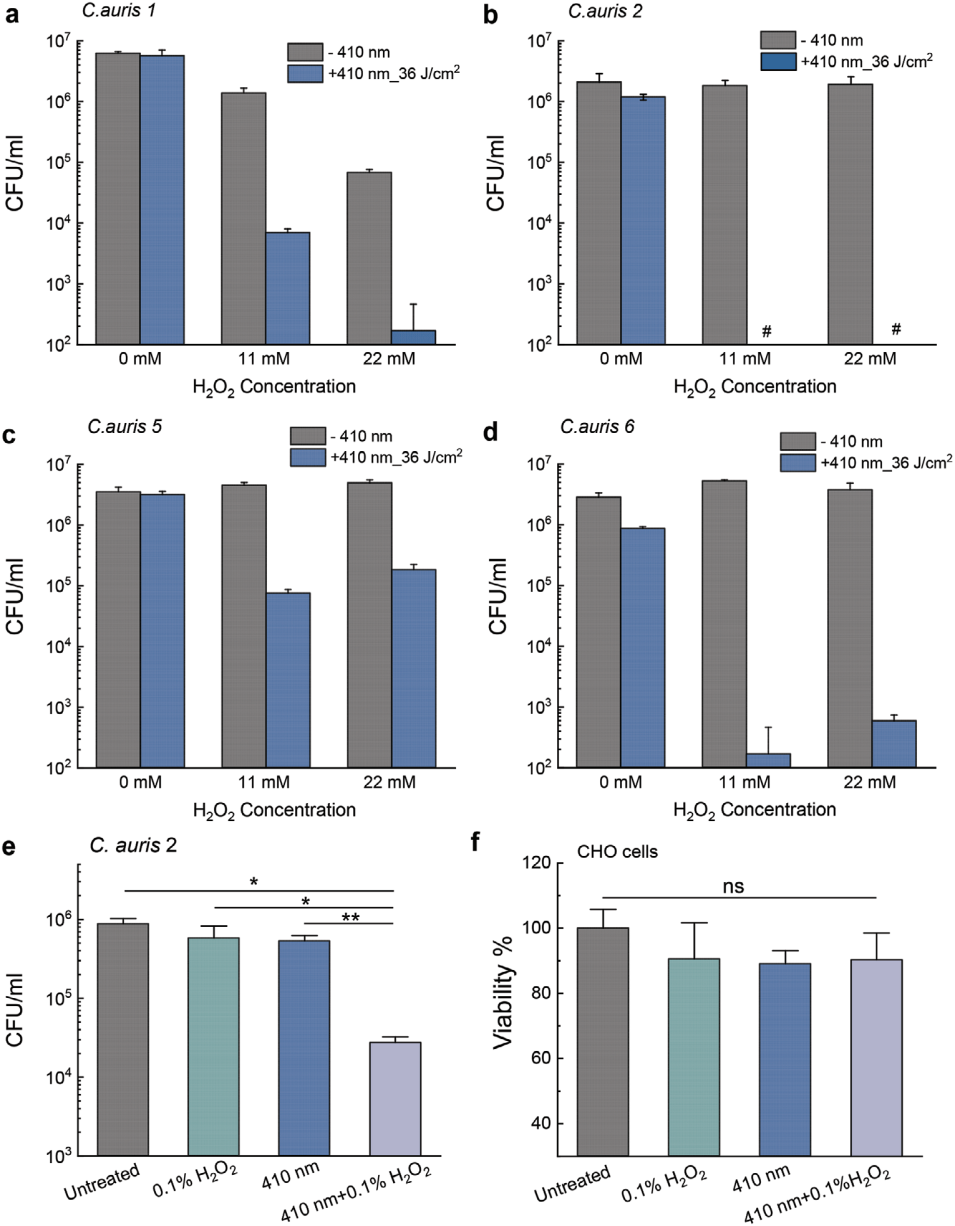
Elimination of various clinical *Candida auris* strains via synergy between photoinactivation of catalase and H_2_O_2_. CFU/mL of log‐phase a) *C. auris 1*, b) *C. auris 2*, c) *C. auris 5*, and d) *C. auris 6* under different treatment schemes. Data: Mean + SD. *N* = 3. Pound sign (#) means below the detection limit. H_2_O_2_‐incubation time: 4 h. 410 nm: 36 J cm^−2^. e) CFU/mL of *C. auris 2* under 410 nm blue light followed by short‐time incubation with 0.1% H_2_O_2_ (30 × 10^−3^ m, 1 min incubation). 410 nm blue light dose: 30 J cm^−2^. f) Viability of CHO cells by MTT assay under the same treatment conditions as panel I. Statistical analysis was achieved by Student unpaired *t*‐test. **: *p* < 0.01; *: *p* < 0.05; ns: not significant.

Log‐phase *C. auris* isolates were exposed to 410 nm blue light (36 J cm^−2^) followed by subsequent administration of H_2_O_2_ (4 h incubation at 30 °C, 11–22 × 10^−3^ m), serial dilution and CFU enumeration. As shown in **Figure** **4**a, in the case of *C. auris 1*, photoinactivation of catalase renders this pathogen highly susceptible to H_2_O_2_ by around three orders of magnitude. Drastically, total eradication of *C. auris 2* was obtained in the 410 nm plus H_2_O_2_ treated group (Figure 4b). Similar augmentation effect appeared regarding *C. auris 5* and *C. auris 6* as well (Figure 4c,d). These results underline the essential role of catalase for *C. auris* to defend against external H_2_O_2_ attack. Collectively, photoinactivation of catalase works synergistically with H_2_O_2_ against clinical *C. auris*.

It was reported that hydrogen peroxide vapor was utilized as clinical room disinfection in order to get rid of *C. auris* colonization,^[^ ^39^ ^]^ however the *C. auris* biofilm is difficult to eradicate explaining the concerning epidemiology of high relapse rates in clinical settings.^[^ ^38^ ^]^ To query the clinical potential of this synergistic therapy which requires shortened H_2_O_2_ treatment time, we then reduced both the H_2_O_2_ incubation time and 410 nm blue light treatment time, and tested if such synergistic treatment remains effective against *C. auris*. We treated 410 nm blue light‐exposed (2.5 min) *C. auris 2* with H_2_O_2_ at a higher concentration (30 × 10^−3^ m, 0.1%) for only 1 min, then followed by CFU enumeration. As shown in Figure 4e, there was no significant difference between the untreated group and either blue light alone‐treated group or H_2_O_2_ alone‐treated group. However, around 99% of fungal burden (Figure 4e) was reduced once combining these two treatments together, suggesting the consistent and effective synergy between photoinactivation of catalase and H_2_O_2_. Noteworthy, the same treatment schemes did not exert detrimental effects on a Chinese hamster ovary (CHO) cell line according to MTT assay (Figure 4f). Collectively, photoinactivation of catalase and H_2_O_2_ exhibit synergistic fungicidal effects against multiple clinical *C. auris* strains with no apparent toxicity to mammalian cells.

### 2.5 Proliferation Inhibition of Wide‐Ranging *C. auris* Strains by Combining Photoinactivation of Catalase with ROS‐Producing Agents

Due to the prominent significance of *C. auris*‐caused breakouts, we particularly investigated whether photoinactivation of catalase could sensitize wide‐ranging *C. auris* strains to multiple ROS‐producing agents (H_2_O_2_, amphotericin B, fluconazole) in a high‐throughput approach, PrestoBlue proliferation assay was utilized again to investigate the fungistatic effects under various treatment schemes (Figures S7–S11, Supporting Information). Meanwhile, time‐course PrestoBlue fluorescence intensity of untreated fungal cells with known CFU number were recorded as standard curves. In this way, we could derive the relatively proliferated fungal cell number from the treated groups based on the linear calibration curves (Figures S12 and S13, Supporting Information).

**Figure 5:**
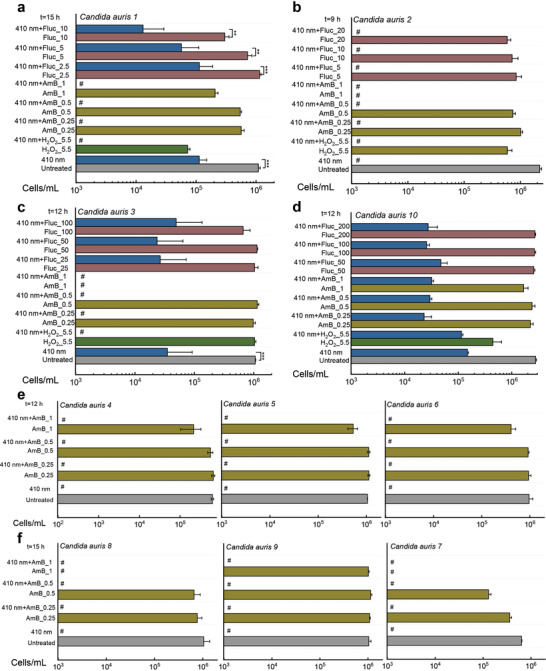
Derived cells/ml from ten clinical *Candida auris* strains based on the PrestoBlue proliferation assay and calibration curves. Data: Mean + SD. *N* = 3. Abbreviations: amphotericin B (AmB), fluconazole (Fluc), unit: µg mL^−1^. Significant different was determined through Student unpaired *t*‐test. *: *p* < 0.05, **: *p* < 0.01; ***: *p* < 0.001. Pound sign (#) means calculated values are below the detection limit.

As shown in **Figure** **5**a,b, 410 nm blue light alone (30 J cm^−2^) reduces the proliferation of *C. auris 1* by around one log_10_, and blue light alone (30 J cm^−2^) significantly suppressed the growth of *C. auris 2*. Total inhibition effect was obtained in the 410 nm plus H_2_O_2_ (5.5 × 10^−3^ m) treated group. Meanwhile, we also observed that total inhibition of proliferation happened in the 410 nm plus amphotericin B (AmB) treated group whereas AmB alone exerted limited efficacy. Noteworthy, photoinactivation of catalase could enhance the fungistatic effects of fluconazole by around one order of magnitude (Figure 5a). Similar phenomena were observed in the case of *C. auris 3* (MIC of fluconazole is 128 µg mL^−1^) and *C. auris 10* (MIC of fluconazole is >256 µg mL^−1^) as well (Figure 5c,d). Strikingly, we further found that multiple *C. auris* strains are highly susceptible to 410 nm blue light treatment alone (Figure 5e,f) as total inhibition was observed, hinting that multiple blue light‐sensitive endogenous chromophores existing inside *C. auris* strains. Collectively, *C. auris* were highly sensitive toward blue light, and photoinactivation of catalase renders *C. auris* susceptible to various ROS‐producing agents, even for some antifungal agents to which *C. auris* have developed resistance.

### 2.6 Photoinactivation of Catalase Boosts Macrophage Killing of *C. albicans*

When fungal infection occurs, pathogenic fungi will encounter our critical line of defense, the innate immune system where phagocytic macrophages can recognize, engulf, and destroy these fungal cells.^[^ ^40^ ^]^ It was reported that *C. albicans* can cause macrophage membrane rupture and lysis through hyphal germination.^[^ ^41^ ^]^ Intracellular *C. albicans* can survive and duplicate within human macrophages by harnessing array of strategies. One of such essential strategies is the expression of ROS scavenging enzymes, catalase and superoxide dismutase.^[^ ^40^ ^]^ Thus, we wondered whether photoinactivation of catalase could deprive *C. albicans* off this vital virulence factor, thus facilitating macrophage killing.

**Figure 6:**
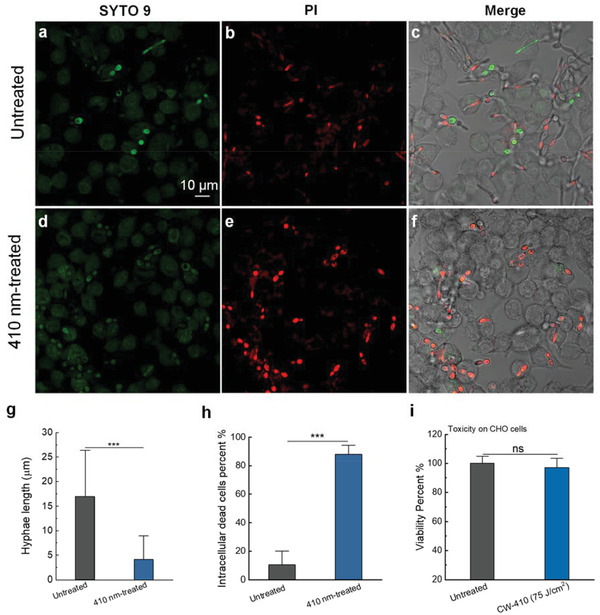
Elimination of intracellular *C. albicans* SC5314 by synergizing photoinactivation of catalase with host innate immune cells. a–f) Confocal imaging of live/dead *C. albicans* SC5314 after *C. albicans* SC5314 (a–c) infection of macrophages RAW 264.7 with (d–f) or without 410 nm blue light pretreatment for 1 h at a multiplicity of infection of 10. g) Quantitative analysis of *Candida* hyphae length of the above two groups. h) Quantitative analysis of intracellular dead bacteria percent of the above two groups. i) CHO cell viability with or without 410 nm blue light treatment by MTT assay. Data: Mean + SD. *N* = 3. Student unpaired *t*‐test. *: *p* < 0.05, **: *p* < 0.01; ***: *p* < 0.001.

To test this hypothesis, we infected a macrophage cell line RAW 264.7 with untreated *C. albicans* and 410 nm blue light‐treated *C. albicans* at a multiplicity of infection of 10 for 1 h. After that, we performed a Live (SYTO 9)/Dead (PI) staining assay to visualize intracellular *C. albicans*. As shown in **Figure** **6**a–c, after 1 h of infection, *C. albicans* indeed exhibited hyphal morphology, and pierced through the macrophages. A small portion of *C. albicans* remained alive inside the macrophages (Figure 6a). By comparison, in the 410 nm blue light‐treated group, we not only observed significantly less live *C. albicans* in their hyphal form, but also significantly shortened hyphal length (Figure 6d,f). Quantitative analysis of the hyphal length under different treatment groups (Figure 6g) further consolidated this finding. Furthermore, there is a significant reduction of *C. albicans* viability after macrophage phagocytosed 410 nm pre‐exposed *C. albicans* (Figure 6h). Collectively, this evidence suggests that photoinactivation of catalase attenuates the virulence of *C. albicans*, thus boosting macrophage elimination of intracellular *C. albicans*. Of note, blue light (410 nm) treatment did not pose significant toxicity to CHO cells (Figure 6i) under the same dosage.

It was also reported that catalase is an indispensable enzyme for *C. albicans* hyphal formation.^[^ ^42^ ^]^ Therefore, there are two plausible explanations for the photoinactivation of catalase‐mediated macrophage killing. On the one hand, photoinactivation of catalase might inhibit hyphal formation through catalase depletion, thus causing less macrophage rupture and lysis; one the other hand, ROS from macrophages can efficiently exert its antimicrobial effect against intracellular *C. albicans* without the protection of catalase. In short, photoinactivation of catalase holds the clinical potential for eliminating intracellular fungal cells.

### 2.7 Photoinactivation of Catalase Reduces Fungal Burden in a *C. albicans*‐Induced Mouse Abrasion Model

**Figure 7:**
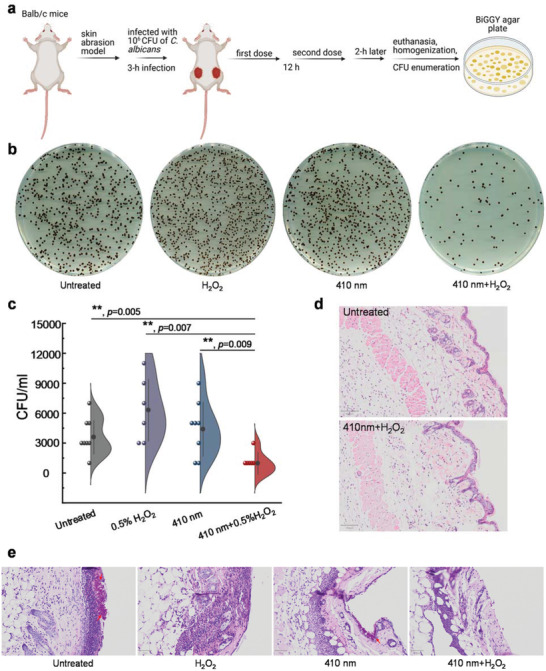
Photoinactivation of catalase and H_2_O_2_ synergistically reduce *C. albicans* burden in a mice skin abrasion model. a) Schematic illustration of development and subsequent treatment for *C. albicans*‐induced mice skin abrasion. b) Spread BiGGY agar plates (*Candida* specific) from homogenized mice wounds after different treatment schemes. c) *C. albicans* CFU/mL of the homogenized mice wounds after different treatments in (a). d) H&E stained histology slides of the untreated mice skin and 410 nm blue light plus H_2_O_2_ treated mice skin. Scalar bar: 100 µm. e) H&E stained histology slides of *C. albicans* infected mice skins under different treatment schemes. Scalar bar: 50 µm. Fungal colonization area was pointed by red arrows. Data: Mean + SE from at least six replicates. Significant difference was determined through Student unpaired *t*‐test. *: *p* < 0.05, **: *p* < 0.01; ***: *p* < 0.001. Outlier was determined through whisker box plot analysis.

With these in vitro implications, we query the clinical utilization of photoinactivation of catalase against fungal infections. It was reported that *C. albicans*‐caused infections usually start from superficial infections before developing severe systematic infections.^[^ ^43^ ^]^ Therefore, we chose a *C. albicans* infected mouse skin abrasion model^[^ ^44^ ^]^ to evaluate the treatment efficacy in vivo. Briefly, 10^6^ CFU of *C. albicans* SC5314 was applied to abraded mouse skin for 3 h, after that different treatment schemes (120 J cm^−2^, 410 nm blue light, 0.5% H_2_O_2_, 410 nm blue light plus 0.5% H_2_O_2_) were then administered to the infected wounds (**Figure** **7**a). Two hours after the second dose, mice were euthanized, and infected wounds homogenized and serially diluted onto *C. albicans*‐specific BiGGY agar plates.^[^ ^45^ ^]^

As shown in Figure 7b, *C. albicans* isolated from infected wounds presented as smooth black colonies on the BiGGY agar plates. Under the same dilution factor, 410 nm plus 0.5% H_2_O_2_ treated group apparently showed the lowest number of *C. albicans* colonies compared to the other treatment groups. Quantitative analysis of CFU/mL from homogenized wounds further confirmed that the fungal burden in the 410 nm blue light plus 0.5% H_2_O_2_ treated group was consistently the lowest compared to the other three groups (Figure 7c). These results suggest that photoinactivation of catalase is effective in reducing overall fungal burden in the clinical‐relevant settings.

To interrogate whether the above treatment induces skin damage, we further examined and compared the normal mouse skin with or without 410 nm blue light (120 J cm^−2^) plus H_2_O_2_ treatment through hematoxylin and eosin (H&E) staining analysis. As shown in Figure 7d, epidermis, dermis, and subcutaneous tissues from the treated mouse skin appeared as intact as that of untreated one, indicating that there is no detectable toxicity from the 410 nm blue light (120 J cm^−2^) plus H_2_O_2_ (0.5%) treatment.

Meanwhile, we also employed H&E staining assay to visualize *C. albicans* infected mice skin from the above treatment groups. Yeast‐form *C. albicans* turned into hyphal structures after 17 h of infection (Figure 7e), consistent with the fact that *C. albicans* usually transitions from yeast to hyphae form in disease settings.^[^ ^46^ ^]^ Of note, neutrophils or macrophages infiltration likely happened in the area adjacent to fungal infection site based on the histology analysis, further immunohistochemistry staining analysis is needed to understand which specific immune cells phagocytosed *C. albicans*. Strikingly, in the groups involved with 410 nm blue light treatment, hyphae‐form *C. albicans* barely showed up. Only few *C. albicans* remained on the mice skin surface after 410 nm plus 0.5% H_2_O_2_ treatment. Collectively, photoinactivation of catalase reduces the virulence of *C. albicans*, thus rendering this pathogen highly susceptible to exogenous H_2_O_2_ attack in a clinically relevant mice fungal infection model.

### 2.8 Consecutive Blue Light Treatment Does Not Induce Resistance Development by *C. albicans*

**Figure 8:**
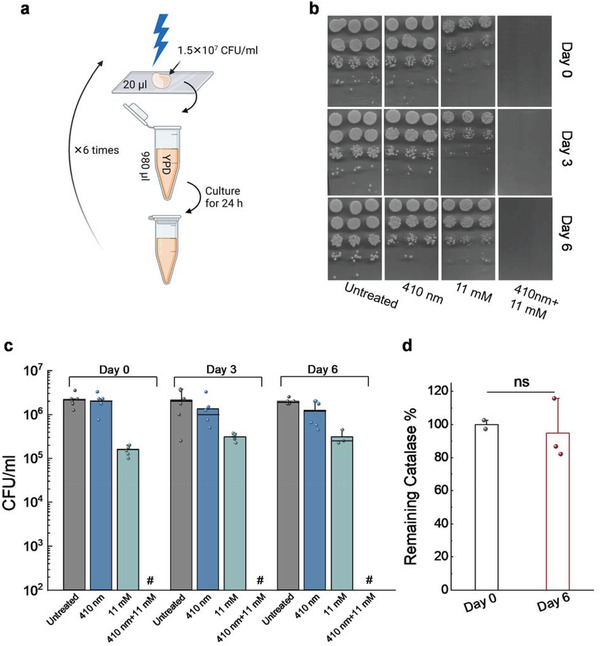
*Candida albicans* does not develop resistance toward consecutive blue light treatments. a) Schematic illustration of the serial blue light treatment and passage experiment. b) Spread plates of CFU of *C. albicans* from Day 0, 3, 6 under different treatment schemes. c) Quantitative analysis of *C. albicans* CFU/mL under various treatments. Pound sign (#) indicates the CFU results are below the detection limit. d) Catalase amount comparison from *C. albicans* before and after six‐consecutive blue light exposure. Student unpaired *t*‐test. ns: not significant. 410 nm: 60 J cm^−2^.

In most of clinical settings, multiple doses of treatments are usually necessary to ensure sufficient clearance of fungal cells. To understand whether continuous 410 nm treatment could induce fungal resistance, we examined the performance of *C. albicans* after six‐consecutive‐day blue light treatment (**Figure** **8**a). First, CFU enumeration assay was performed on *C. albicans* at day 0, 3, 6 under different treatment schemes. Next, we compared the catalase amount of *C. albicans* at day 0 and day 6 through the Amplex red catalase kit.

As shown in Figure 8b, *C. albicans* from day 0, 3, 6 demonstrated similar sensitivity toward H_2_O_2_ treatment, and total eradication of *C. albicans* in the 410 nm blue light plus H_2_O_2_ treated group was achieved consistently. Quantitative analysis of CFU/mL results further consolidated the results, indicating that *C. albicans* does not develop resistance toward consecutive blue light treatments.

To understand whether catalase amount has any difference before and after the consecutive treatments, we compared the catalase amount inside *C. albicans* at day 0 and day 6 using the Amplex red catalase kit. Interestingly, we did not find significant difference between these two groups (Figure 8c), further suggesting that catalase, the molecular target of 410 nm blue light, is consistently expressed throughout the serial passage. And *C. albicans* is unlikely to develop resistance toward 410 nm blue light‐based therapy.

## 3 Discussion

Antifungal resistance has raised alarms and threats to clinicians and patients due to the limited arsenal of antifungal agents as well as current impeded antifungal development pipeline.^[^ ^47^ ^]^ For severely ill or immunocompromised patients, this situation especially worsens, and systematic candidiasis or candidemia might happen. Association of invasive aspergillosis accompanying COVID‐19 patients as secondary infection have been reported.^[^ ^48^ ^]^ In addition, the global overuse of antifungals accelerates development of multidrug or pan‐drug resistant fungal cells. Therefore, novel approaches to combat fungal infection are highly desired. Here, using wild type *C. albicans* along with catalase‐deficient *C. albicans*, we first identified catalase residing inside wide‐ranging fungal species as a primary target of blue light. Its function in detoxifying intracellular H_2_O_2_ can be efficiently inactivated by blue light, especially around 410 nm range, which subsequently renders these fungal species highly susceptible to ROS‐producing agents. In addition, the synergy between photoinactivation of catalase and H_2_O_2_ remains effective for various fungal species and strains, especially, the notorious clinical drug‐resistant *C. auris* strains. Toward clinical applications, we found that photoinactivation of catalase boosts macrophage killing of intracellular *C. albicans* and reduces *C. albicans* burden in a *C. albicans* infected mouse model of skin abrasion once combining with hydrogen peroxide. Consecutive blue light passage study indicates that neither catalase expression level nor cell susceptibility to blue light is changed for serial blue light treatment. These findings suggest the great potential of catalase‐targeting blue light therapy as a new alternative to treat clinical multidrug resistant fungal infections.

Of note, blue light‐mediated inactivation of *C. albicans* has been documented before.^[^ ^11b^ ^]^ However, the underlying molecular target and the eradication mechanism for blue light are still unclear, which seriously hinders follow‐up developments in improving its efficacy as well as its clinical translations. Based on our studies here we unveiled that blue light alone only exerts limited efficacy by inactivating catalase and ROS‐producing agents are essential to be administered as adjuvants to synergize with 410 nm blue light for strikingly improved fungal eradication. For example, the light dose and settings we applied here are well below the ANSI standard (200 mW cm^−2^ for 410 nm), and blue light‐mediated photoinactivation of catalase sensitizes *C. albicans* to nonfungicidal low‐concentration H_2_O_2_ by around six orders of magnitude. As another example, AmB, the golden standard for antimycotic treatment for the most severe fungal infections,^[^ ^49^ ^]^ exhibits part of its killing efficacy through ROS burst after binding with ergosterol.^[^ ^50^ ^]^ However, high‐dose AmB treatment often leads to acute renal failure as a well‐documented serious complication.^[^ ^51^ ^]^ Our findings suggest an alternative approach to tune down the usage of AmB by photoinactivation of catalase. Moving further, photoinactivation of catalase also revives certain antifungal agents such as fluconazole against azole‐resistant clinical *C. auris* strains (Figure 5c,d).

We found that multiple clinical *C. auris* strains are exceptionally sensitive to blue light when compared to the other fungal cells as total growth inhibition was obtained through PrestoBlue proliferation assay (Figure 5). However, it remains enigmatic regarding exactly why *C. auris* has such high sensitivity to blue light. Likely, it possesses relatively higher amount of catalase when compared to other *Candida* species. Nevertheless, further quantitative characterizations of catalase amount and related mRNA expression levels are helpful to address this query. *C. auris* also demonstrates a propensity to persist on skin or environmental surfaces, a unique feature to distinguish it from other *Candida* species, thus highly transmissible.^[^ ^52^ ^]^ Our data here demonstrates the high susceptibility of *C. auris* to blue light, and blue light alone can be developed as a novel approach for eradicating drug‐resistant *C. auris* colonized on skin or environmental surfaces.

We also found that photoinactivation of catalase boosts macrophage killing of intracellular *C. albicans*. Particularly, shortened hyphae length was observed inside the macrophages infected by the 410 nm pre‐exposed *C. albicans*, suggesting the pivotal role of catalase plays in the transition from yeast‐ to hyphae‐form *C. albicans*. It has been reported catalase gene disruption did not induce hyphae formation in yeast‐form *Saccharomyces cerevisiae*.^[^ ^42^ ^]^ Therefore, further molecular‐level study to further understand how 410 nm blue light treatment modulates such yeast‐to‐hyphae transformation can be pursued. In addition, significant number of yeast‐form *C. albicans* were found dead inside macrophages, suggesting an effective 410 nm blue light‐mediated macrophage phagocytosis. Other immune cells like neutrophils also produce high level of ROS when phagocytosing fungal cells.^[^ ^53^ ^]^ Therefore, we suspect that photoinactivation of catalase could assist neutrophils to eliminate intracellular fungal cells via a similar mechanism. Taken together, photoinactivation of catalase presumably enhances immune cells to phagocytose fungal cells, thus preventing the potential disseminated candidiasis.

In an independent and parallel study, we identified catalase in a wide range of bacterial species as a primary molecular target of blue light and further demonstrated the potential utilization of photoinactivation of catalase to eradicate various pathogenic bacteria through combination with H_2_O_2_‐producing agents.^[^ ^54^ ^]^ This work unveiled catalase as a primary target of blue light in wide‐ranging fungal species, thus broadening the scope of utilization of photoinactivation of catalase to an even wider range of pathogenic microbes. Although catalase is identified as the primary target of blue light, we believe that other molecular targets other than catalase might still exist. This is evidenced by enhanced H_2_O_2_ killing of catalase‐deficient *C. albicans* by 410 nm blue light. To identify these peripheral targets in the future, endogenous pigments could be further examined as intrinsic chromophores, e.g., staphyloxanthin, have shown striking sensitivity to blue light‐mediated photobleaching.^[^ ^55^ ^]^ It has also been reported that melanin is the major pigment inside *C. albicans* playing a myriad of biological functions.^[^ ^56^ ^]^ Photoinactivation or photomodulation of melanin might account for blue light‐mediated killing as well. Another direction to pursue is to improve light penetration depth, as blue light currently can only penetrate a few hundred microns,^[^ ^57^ ^]^ thus limiting its applications only to superficial surfaces. For deep‐seated fungal infections, pulsed blue laser with high‐peak power might offer improved and deeper penetration compared to the continuous wave light.^[^ ^55b^ ^]^ Upconverting nanoparticles, capable of converting near‐infrared red excitation into visible and ultraviolet emission,^[^ ^58^ ^]^ might suggest another potential way to treat deep‐tissue fungal infections. In summary, our findings here suggest a fundamental molecular target and eradication mechanism of blue light elimination of wide‐ranging fungal species. These mechanistic insights create new possibilities of finding highly effective treatment avenues for surface sterilization and for treatment of drug‐resistant fungal infections.

## 4 Experimental Section

Blue light source: CW blue light was delivered through a mounted 405 nm blue light LED (M405L4, Thorlabs) with an adjustable collimation adapter (SM2F32‐A, Thorlabs) focusing the illumination region to a ≈1 cm^2^ region. A T‐Cube LED driver (LEDD1B, Thorlabs) allowed for adjustable light fluencies up to 500 mW cm^−2^.

### Fungal Strains and Chemicals

Fungal strains: *Candida albicans* SC5314 (wild type, American Type Culture Collection (ATCC)), and catalase‐deficient *Candida albicans* 2089 (△*cat1*) were from Dr. Alistair Brown lab at University of Exeter. All the other strains were from Dr. Michael K. Mansour's lab (clinical strains from Massachusetts General Hospital (MGH), Boston, MA) including all the *Candida auris* strains: *Candida auris 1*_MGH, *Candida auris 1* (AR‐0381), *Candida auris 2* (AR‐0382), *Candida auris 3* (AR‐0383), *Candida auris 4* (AR‐0384), *Candida auris 5* (AR‐0385), *Candida auris 6* (AR‐0386), *Candida auris 7* (AR‐0387), *Candida auris 8* (AR‐0388), *Candida auris 9* (AR‐0389), *Candida auris 10* (AR‐0390). *Candida albicans C15*, *Candida albicans C16*, *Candida albicans C17*. *C. glabrata* (ATCC 2001), *Candida glabrata C1*, *Candida glabrata* C2, *C. tropicalis* (H3222861), *C. parapsilosis* (F825987), *C. lusitaniae* (S1591976), *C. lusitaniae* (AR‐0398), *C. haemulonii* (AR‐0393), *C. haemulonii* (AR‐0395), *Candida duobushaemulonii* (AR‐0394), and *C. krusei* (AR‐0397). All cell lines used in this study, including the RAW 264.7 murine macrophages, CHO cells were purchased directly from the ATCC.

Chemicals: Amphotericin B (A9528‐100MG, Sigma‐Aldrich), hydrogen peroxide (CVS Health), DMSO (D8418‐500ML, Sigma‐Aldrich), yeast peptone dextrose (YPD) broth (Y1375, Sigma‐Aldrich), YPD Agar (Y1500, Sigma‐Aldrich), phosphate buffered saline (BP399500, Fisher Scientific), 10% formalin (HT501128‐4L, Sigma‐Aldrich), sodium chloride solution (S8776, Sigma‐Aldrich), miconazole nitrate salt (M3512, Sigma‐Aldrich), fluconazole (86386, Acros Organics), PrestoBlue reagent (A13261, ThermoFisher), 3‐Amino‐1,2,4‐triazole (3‐AT, A8056, Sigma‐Aldrich), bovine liver catalase (C1345, Sigma‐Aldrich), LIVE/DEAD cell viability kit (L7007, ThermoFisher), BiGGY agar (73608, Sigma‐Aldrich), Amplex Red Catalase Assay (A22180, Thermo Fisher Scientific), Dulbecco's modified Eagle medium (DMEM) (2186822, Gibco), HI fetal bovine serum (FBS) (2273356P, Gibco), and HEPES (54457, Sigma‐Aldrich).

Fungal culture: *C. albicans* was routinely streaked at 30 °C onto the YPD agar and subcultured in YPD broth overnight to stationary phase. Stationary‐phase *Candida* strains were cultured into mid‐log phase prior use by 1:20 dilution by YPD broth. Fungal cell density was adjusted based on the optical density at 660 nm (OD_600_). The suspension was centrifuged, washed with phosphate‐buffered saline (PBS), and resuspended in PBS at the cell density of 10^6^–10^7^ CFU mL^−1^ before proceeding all the treatments.

Quantitation of remaining catalase percentage: Measurement of remaining catalase percentage was primarily quantified through the use of an Amplex red Catalase Assay (A22180, Thermo Fisher Scientific) according to its standard protocol. Briefly, solutions containing catalase (either bovine liver catalase or catalase‐positive fungal culture with 1 × 10^6^ cell density) were treated with blue light, after which 25 µL of the light treated solution was incubated with 40 × 10^−6^ m of H_2_O_2_ for 2 h at 30 °C in dark inside a 96‐well plate. Following H_2_O_2_ treatment, 50 µL of a reaction stock containing 100 × 10^−6^ m of Amplex Red and 0.4 U mL^−1^ of horseradish peroxidase were added to each well and then incubated for 30 min at 37 °C. Following incubation, the fluorescence of each well was measured at an excitation wavelength of 545 nm and an emission wavelength of 590 nm. A negative control containing the 1× reaction buffer (0.1 m Tris‐HCl) and an untreated positive control (normal fungal cells without blue light exposure) also treated with the assay in order to determine the remaining catalase percentage. Remaining catalase percentage was calculated through the following equation

(2) $$\mathrm{Remaining}\;\mathrm{catalase}\;\%\;=\;\frac{I_{\mathrm{buffer}}-I_{\mathrm{treated}}}{I_{\mathrm{buffer}}-I_{\mathrm{untreated}}}\times \;100\%$$

### Imaging of Intracellular H_2_O_2_

*C. albicans* with and without 410 nm exposure (30 J cm^−2^) were incubated with H_2_O_2_ (22 × 10^−3^ m, 30 min incubation). After that, *C. albicans* were stained with an intracellular H_2_O_2_ kit (MAK164‐1KT) for 30 min. Fungal cells were then washed and sandwiched between a cover slide and poly‐lysine‐coated cover glass. Confocal laser scanning microscopy (FV3000, Olympus) was then applied to image intracellular H_2_O_2_.

Bubbling test, CFU enumeration assay, short‐term incubation assay, checkerboard broth dilution assay, and time killing assay to validate the mechanism of catalase inactivation in catalase inhibited or knocked‐out Candida strains: *Candida albicans* SC5314 and *Candida albicans* △*cat1* were cultured overnight in YPD broth at 30 °C using an orbital shaking incubator (250 rpm).

For the bubbling test, after overnight culturing, both the wild type *C. albicans* SC5314 and *Candida albicans* △*cat1* were washed by PBS and adjust the cell density to 10^7^ mL^−1^, placed 1 mL of fungal solution into cuvettes with 80 µL of 3% H_2_O_2_, and then incubated at room temperature for 30 min before observation.

For the CFU enumeration, the next day after overnight culture, fungal cultures were resuspended 1:20 in YPD for another 5–6 h and cultured into mid‐log phase. Then fungal cells were washed, suspended in PBS and adjusted at the optical density by 600 nm (OD_600_) to ≈10^8^ CFU mL^−1^, after which a 10 µL of aliquot was placed on a glass cover slide and then treated with CW‐410 nm (100 mW cm^−2^, 5 min). Following light treatment, the droplet was collected to a tube containing PBS or PBS supplemented with H_2_O_2_. H_2_O_2_ ranged from 0.69 to 22 × 10^−3^ m according to the sensitivity of fungal strains. Samples were then incubated for 30 min at 30 °C in the orbital shaking incubator, after which the treated and untreated fungal samples were tenfold serially diluted inside a 96‐well plate, plated on YPD agar plates in a 30 °C static incubator for 24–36 h prior to enumeration. All the CFU results were read at the same time for each patch of experiment.

Short‐term incubation assay: In order to determine the impact of short term H_2_O_2_ exposure on *C. auris* following light induced catalase inactivation, *C. auris* 2 was incubated overnight in YPD broth. The next day 200 µL of *C. auris* was centrifuged and resuspended in 1 mL of 1 × PBS. A 10 µL aliquot of *C. auris* suspension was placed on a glass coverslip and exposed to 30 J cm^−2^ of CW‐410 (200 mW cm^−2^, 2.5 min). Following exposure, the aliquot was transferred to 990 µL of PBS and vortexed. This process was repeated for the nonlight treated *C. auris* as well. After the creation of the light treated and untreated *C. auris* stock, 300 µL of stock was separated and treated with 0.1% H_2_O_2_ for 1 min, after which the H_2_O_2_ treated suspension was serially diluted and plated to determine changes in overall CFU following exposure.

For the catalase inhibition assay, 3‐Amino‐1,2,4‐triazole (3‐AT) was applied to both untreated and H_2_O_2_ containing groups at the concentration 50 × 10^−3^ m), and cultured with *C. albicans* SC5314 for 4 h in a 30 °C shaking incubator. After treatment, the treated suspensions were spun down and resuspended in fresh PBS, after which a 10 µL aliquot of untreated *C. albicans* was treated with 30 J cm^−2^ of CW‐410 (100 mW cm^−2^, 5 min) and then diluted in 990 µL of PBS. Alongside the light treated dilution, an untreated dilution and 3‐AT treated dilution were also produced. These dilutions were treated with 44 × 10^−3^ m of H_2_O_2_ for 2 h at 30 °C, after which the samples were serially diluted and plated to quantify the overall CFU enumeration.

For the time killing assay, *Candida albicans* SC5314 and *Candida albicans* △*cat1* were cultured into mid‐exponential phase in YPD broth at 30 °C using an orbital shaking incubator (250 rpm). Then the fungal culture was washed and adjusted based on OD_600_ to ≈10^8^ CFU mL^−1^, after which a 10 µL aliquot was placed on a glass cover slide and then treated with CW‐410 nm (100 mW cm^−2^, 5 min). Following light treatment, the droplet was collected to a tube containing PBS or PBS containing 11 × 10^−3^ m H_2_O_2_. Samples were shaken and incubated at 30 °C for up to 4 h. During the incubation, 60 µL aliquots were removed from each sample tube for serial dilution and CFU plating at the 0 min, 20 min, 40 min, 1 h, 1.5 h, 2 h, and 4 h time points.

For the checkerboard assay, *Candida albicans* SC5314 and *Candida albicans* △*cat1* were cultured into mid‐exponential phase in YPD broth. Then fungal culture was washed and adjusted based on OD_600_ to ≈10^7^ CFU mL^−1^ as stock solution and stored on ice. An aliquot of 5 µL fungal stock solution was exposed with 410 nm light at a power of 500 mW cm^−2^ for 0 min, 2.5 min, 5 min, 10 min, 20 min, 40 min, then collected with 995 µL of YPD broth to a final cell density of ≈5 × 10^4^ mL^−1^ and plated into a 96‐well plate in row. Then a twofold serial dilution was performed from 22 × 10^−3^ m of H_2_O_2_ to the following concentrations: 11 × 10^−3^, 5.5 × 10^−3^, 2.8 × 10^−3^, 1.4 × 10^−3^, 0.7 × 10^−3^, 0.4 × 10^−3^, 0.2 × 10^−3^, 0 × 10^−3^ m. Time‐course OD_600_ was recorded by the plate reader over 18 h. The combinational behavior between blue light and H_2_O_2_ from both wild type *C. albicans* and catalase mutant was then calculated based on the equation shown in this paper.

### Time‐Course PrestoBlue Proliferation Assay of Log Phased and Stationary Phased *Candida* Species

Clinical fungal strains from MGH Dr. Michael K. Mansour's lab were used in this part to test the broad band of the phototherapy, and a high throughput resazurin‐based PrestoBlue assay was applied. Both stationary‐ and exponential phase fungal cells were included as well. Stationary phase *Candida* species were prepared by overnight culturing at 30 °C using an orbital shaking incubator (250 rpm); and the log phase fungal cells were prepared the next day by 1:20 in YPD for another 5–6 h and cultured into mid‐log phase, some of the *C. auris* strains took longer time to grow. All cells were collected and washed twice, then the cell density was adjusted to ≈10^8^ mL^−1^ prior to blue light exposure. A 10 µL of aliquot was placed onto a glass cover slide and then treated with CW‐410 nm (100 mW cm^−2^, 5 min) or not. Following light treatment, the droplet was collected to a tube containing 990 µL of PBS to reach the fungal cell density of ≈10^6^ mL^−1^. H_2_O_2_ ranging from 11 × 10^−3^to 44 × 10^−3^ m was added to the wells in the first row and a twofold dilution was conducted, the initial concentrations were chosen according to the sensitivity of fungi strains. Samples were then incubated for 4 h at 30 °C, after which an aliquot of 90 µL of YPD and 10 µL of PrestoBlue reagent were added to each well, followed by a time‐course recording of the fluorescence signal (Excitation: 590 nm) for 24 h. Calibration growth curves with different initial cell density was also conducted in order to calculate the original CFU of all the *Candida* strains.

### Intracellular Fungi Assay

To assess the fungal intracellular killing with murine macrophage RAW 264.7 cell line, *C. albicans* SC5314 was collected from an exponentially growing culture and washed with PBS. Macrophages were cultured in DMEM alongside 10% FBS until 90% confluence was achieved. Macrophages were prewashed with serum‐free DMEM media immediately before infection and infected by *C. albicans* SC5314 (MOI = 10) with and without 410 nm treatment (35 mW cm^−2^, 8 min). Then coculture was incubated at 37 °C in a humidified incubator to allow for the phagocytosis of fungal cells. After 2 h, the infection coculture was removed and replaced with normal growth media (DMEM supplemented with 10% FBS, 10 × 10^−3^ m HEPES). A Live (SYTO 9)/Dead (PI) staining assay was to utilize to visualize the live and dead fungal cells inside macrophages, respectively. Briefly, after fixation with 10% formalin following infections, samples were permeabilized with 0.1% Triton‐X for 3 min at room temperature. After that, a Live/Dead fluorescence kit (Thermo Fisher Scientific, L7007) was utilized to stain the intracellular fungal cells and confocal laser scanning microscope (FV3000, Olympus) was employed to visualize the stained samples.

Mammalian cell toxicity assay: To evaluate the potential toxicity of 410 nm exposure and short term, high concentration H_2_O_2_ exposure against mammalian cells, an MTT assay was performed using CHO cell line. CHO cells were cultured in DMEM and 10% FBS until a high confluence was obtained. CHO cells were then removed via trypsin, quantified, and diluted in serum‐free DMEM media to a cell concentration of 1 × 10^6^ cells mL^−1^. In a treated 96‐well plate, 100 µL of cell suspension was added to each well, providing 1 × 10^5^ cells per well. The cells were allowed to adhere overnight at 37 °C with 5% CO_2_.

The next day, the media was removed from each well and the cells were washed twice with PBS to minimize potential reactions between the phenol red present in DMEM and the blue light treatment. Washing conditions performed on one set of cells were also performed on all other sets to maintain consistency. Following washing, all wells were filled with PBS and the light treated wells were then exposed to 30 J cm^−2^ of CW‐410 (200 mW cm^−2^, 2.5 min) After light treatment, PBS was removed and replaced with DMEM. For the H_2_O_2_ treated groups, 0.1% H_2_O_2_ in DMEM was added to the H_2_O_2_ treatment groups for 1 min, after which the media was immediately removed, and the wells were washed three times with PBS to minimize potential remaining H_2_O_2_ remaining. Threefold washing was also applied to the other treatment groups. After washing, the wells were filled with 100 µL of fresh DMEM and the cells were allowed to recover from treatment over the course of 24 h at 37 °C with 5% CO_2_. Once 24 h have passed, an MTT viability assay was performed, where the DMEM was replaced with fresh DMEM alongside 0.5 mg mL^−1^ of MTT (Life Technologies, M6494) and incubated at 37 °C for 4 h. Once upon completion, the DMEM/MTT solution was removed and 100 µL of filtered DMSO was mixed in and provided with 45 min to dissolve the crystalized formazan, after which absorbance measurements were quantified via plate reader at 590 nm. The assay was performed in replicates of *N* = 4.

In vivo murine infection model and histology: Animal studies were approved by the Institutional Animal Care and Use Committee at Boston University and were performed in the Animal Science Center in the Charles River Campus. The abrasion fungal infection model was used. Twenty BALB/c mice (Jackson Laboratories, 000651) were shaved on the dorsal side and placed under anesthesia, then a sterile #15 scalpel was used to generate two ≈1 × 1 cm^2^ abrasion wounds on both left and right by carefully scraping the epidermis of the skin until reddish dots appeared without drawing blood out of skin barrier. Once wounds were generated, a 10 µL aliquot containing 2 × 10^8^ mL^−1^ of exponential phase *C. albicans* SC5314 in PBS was placed onto the abrasion wound, and spread evenly across the wound with a pipette tip and air dried. Then 20 mice were divided into four treatment groups, each consisting of 5 mice (*N* = 10 wounds in total): Untreated, 410 nm treated, H_2_O_2_ treated, and 410 nm plus H_2_O_2_ treated. Light treatment was applied by positioning mice under 200 mW cm^−2^ (ANSI standard for human skin) 410 nm LEDs for 10 min (total 120 J cm^−2^, 5 min exposure and 5 min break and then 5 min exposure again in order to prevent potential photodamage). For H_2_O_2_ treatment, 10 µL of 0.5% H_2_O_2_ was evenly distributed on the infected wounds and air dried. Combinational treatment consisted of the application of previously described blue light treatment (120 J cm^−2^) followed immediately by the administration of H_2_O_2_. Treatments were applied to mice twice, the first treatment being applied 3 h following infection, and the second treatment being applied 15 h after the infection. 2 h after the second treatment, mice were euthanized and wound tissue was harvested, either homogenized and serially diluted for CFU studies or fixation by 10% neutral buffered formalin for histology studies. CFU enumeration was performed on *C. albicans*‐specific BiGGY agar plates (73608, Sigma‐Aldrich).

The potential phototoxicity of the treatment on the skin was evaluated by H&E histology assay. Same assay was applied as well to examine the location of fungal cells in the infection site. The untreated mouse skin received the same two treatments as the wound site, and during tissue harvesting, this region was excised and preserved in 10% buffered formalin. Formalin fixed samples for both untreated and treated normal skin tissues (*N* = 6) and fungi infected skin tissues were submitted to the Boston Medical Center for histology processing, periodic acid‐Schiff staining for fungal infected skin and H&E staining for normal skin. Histology slides were then visualized by Olympics VS120 slide scanner provided by Micro/Nano Imaging lab in Biomedical Engineering Core Facilities under in Boston University, and then the images were visualized and analyzed by a pathologist Dr. Ivy Rosales at MGH.

### Statistical Analysis

Statistical analysis was conducted through Student unpaired *t*‐test and One‐way ANOVA in R. ***: significantly different with the *p*‐value < 0.001. **: significantly different with the *p*‐value < 0.01. *: significantly different with the *p*‐value < 0.05. ns means no significance. Outlier was determined through whisker box plot analysis.

## Conflict of Interest

The authors declare no conflict of interest.

## Author Contributions

P.‐T.D., Y.Z., S.J., and J.H. contributed equally to this work. P.‐T.D. and J.‐X.C. conceived the synergistic therapeutic treatment between photoinactivation of catalase and H_2_O_2_ or certain antifungals. M.K.M. provided all the clinical fungal strains including the *Candida auris* strains and experimental discussions. P.‐T.D. and J.H. discovered that catalase from catalase‐positive fungi could be ubiquitously inactivated by 410 nm. P.‐T.D. characterized catalase photoinactivation and intracellular fungi assay. P.‐T.D. and Y.Z. conducted the mechanism study by the catalase‐deficient *C. albicans* strains. P.‐T.D. and S.J. conducted the 3‐AT study. P.‐T.D., Y.Z., and Z.D. prepared the clinical fungal samples together. P.‐T.D., Y.Z., and J.H. conducted all the in vitro PrestoBlue assay. P.‐T.D., Y.Z., and S.J. did data analysis of Prestoblue assay. P.‐T.D., Y.Z., and S.J. conducted the in vivo mice abrasion experiments and histology assay. Y.Z. conducted the histology slides scanning and preliminary analysis. P.‐T.D. and J.‐X.C. co‐wrote the manuscript. All authors read and commented on the manuscript.

## Supporting information

Supporting Information

## Data Availability

The data that support the findings of this study are available from the corresponding author upon reasonable request.
