# Supplementary material for: Photoinactivation of Catalase Sensitizes Candida albicans and Candida auris to ROS‐Producing Agents and Immune Cells
Source: Adv Sci (Weinh). 2022 Feb 4;9(10):2104384. doi: 10.1002/advs.202104384 (PMC8981478; doi:10.1002/advs.202104384)
Supplement: Supplementary file 1 — Supporting Information [file ADVS-9-2104384-s001.pdf]

WILEY-VCH

ADVANCED SCIENCE
Open Access

Supporting Information

for *Adv. Sci.*, DOI: 10.1002/advs.202104384

Photoinactivation of catalase sensitizes *Candida albicans* and *Candida auris* to ROS-producing agents and immune cells

*Pu-Ting Dong, Yuewei Zhan, Sebastian Jusuf, Jie Hui, Zeina Dagher, Michael K. Mansour**, and *Ji-Xin Cheng**

Supplementary information for manuscript

Photoinactivation of catalase sensitizes *Candida albicans* and *Candida auris* to ROS-producing agents and immune cells

Pu-Ting Dong[1,2,#], Yuewei Zhan[1,2,#], Sebastian Jusuf[1,2,#], Jie Hui[1,2,#], Zeina Dagher[3,4], Michael K. Mansour[3,4,*], Ji-Xin Cheng[1,2,*]

[1]Department of Biomedical Engineering, Boston University, Boston, MA02215, USA.

[2]Photonics Center, Boston University, Boston, MA02215, USA.

[3]Division of Infectious Diseases, Massachusetts General Hospital, Boston, MA02114, USA.

[4]Harvard Medical School, Boston, MA02115, USA.

*Corresponding authors. Emails: jxcheng@bu.edu and mkmansour@partners.org

[#]These authors contributed equally: Pu-Ting Dong, Yuewei Zhan, Sebastian Jusuf, Jie Hui.

This file includes:

Supplementary Figures 1-13

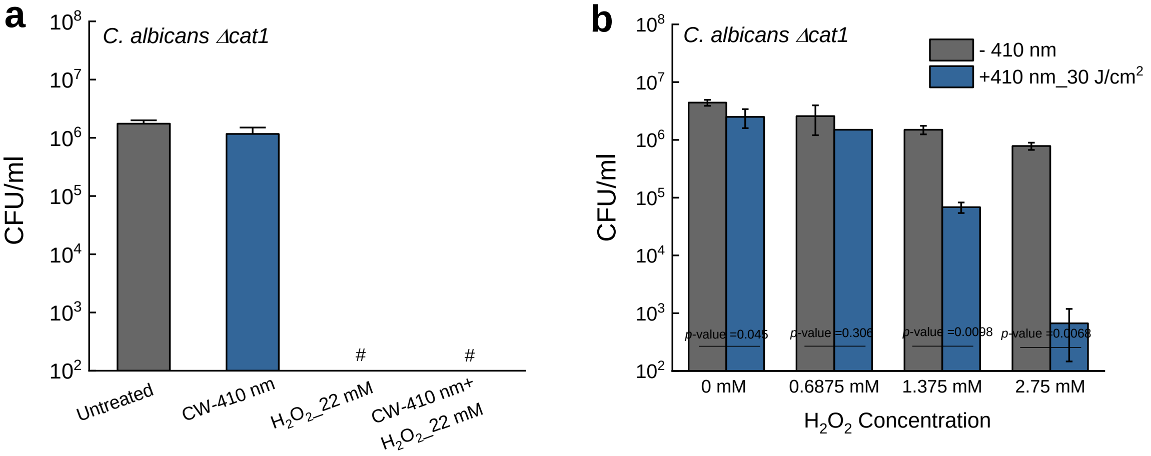

Supplementary Figure 1. CFU/ml of catalase-deficient *C. albicans Δcat1* under different treatment schemes. Data: Mean+SD. N=3. Student unpaired *t*-test. *: $p<0.05$, **: $p<0.01$; ***: $p<0.001$.

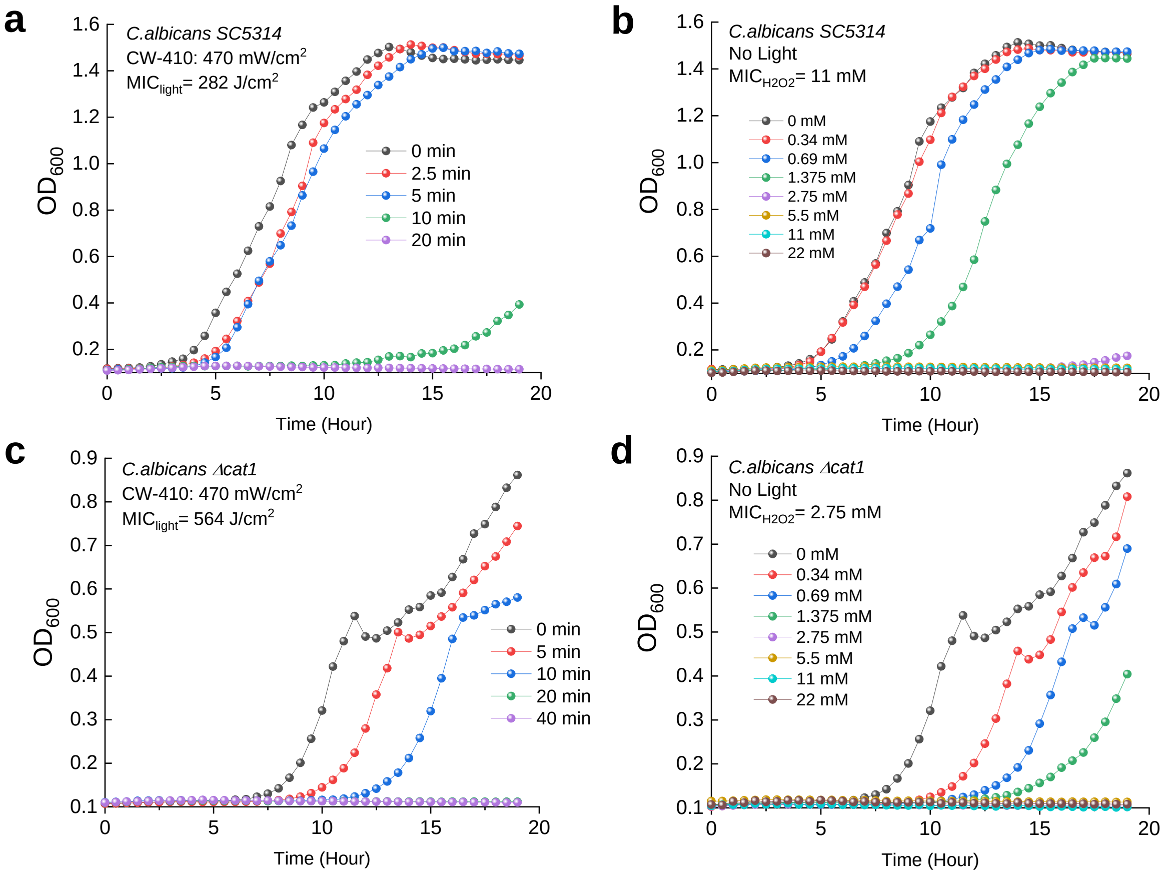

Supplementary Figure 2. Minimal inhibitory concentrations of 410 nm exposure and H_2O_2 against *C. albicans* SC5314 and catalase-deficient *C. albicans Δcat1*.

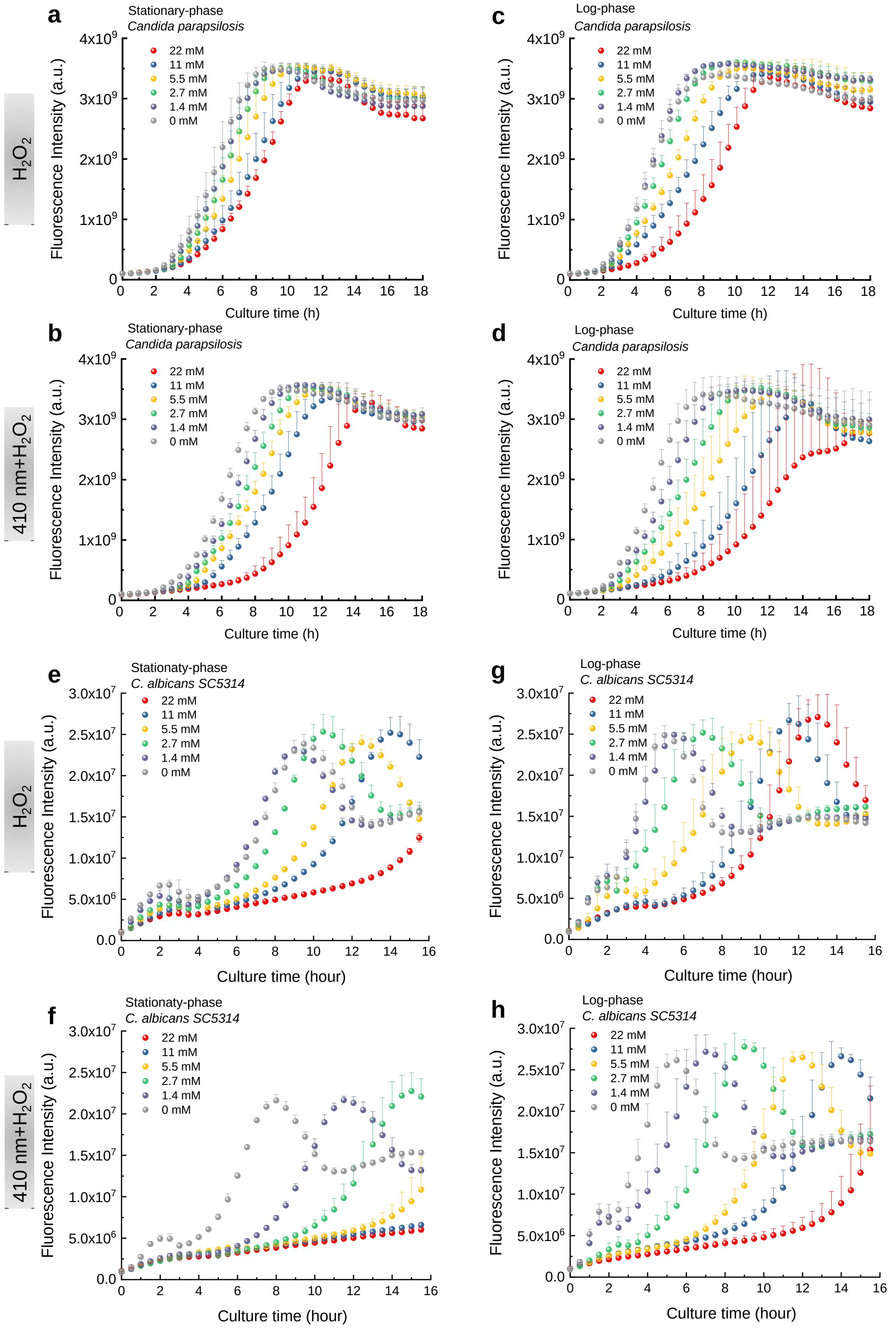
a
Stationary-phase
Candida parapsilosis
b
Stationary-phase
Candida parapsilosis
c
Log-phase
Candida parapsilosis
d
Log-phase
Candida parapsilosis
e
Stationaty-phase
C. albicans SC5314
f
Stationaty-phase
C. albicans SC5314
g
Log-phase
C. albicans SC5314
h
Log-phase
C. albicans SC5314
H2O2
410 nm+H2O2
22 mM
11 mM
5.5 mM
2.7 mM
1.4 mM
0 mM
Fluorescence Intensity (a.u.)
Culture time (h)
Culture time (hour)

Supplementary Figure 3. Time-course PrestoBlue fluorescence intensity from various fungal species under the treatment of 410 nm and H_2O_2 of various concentrations.

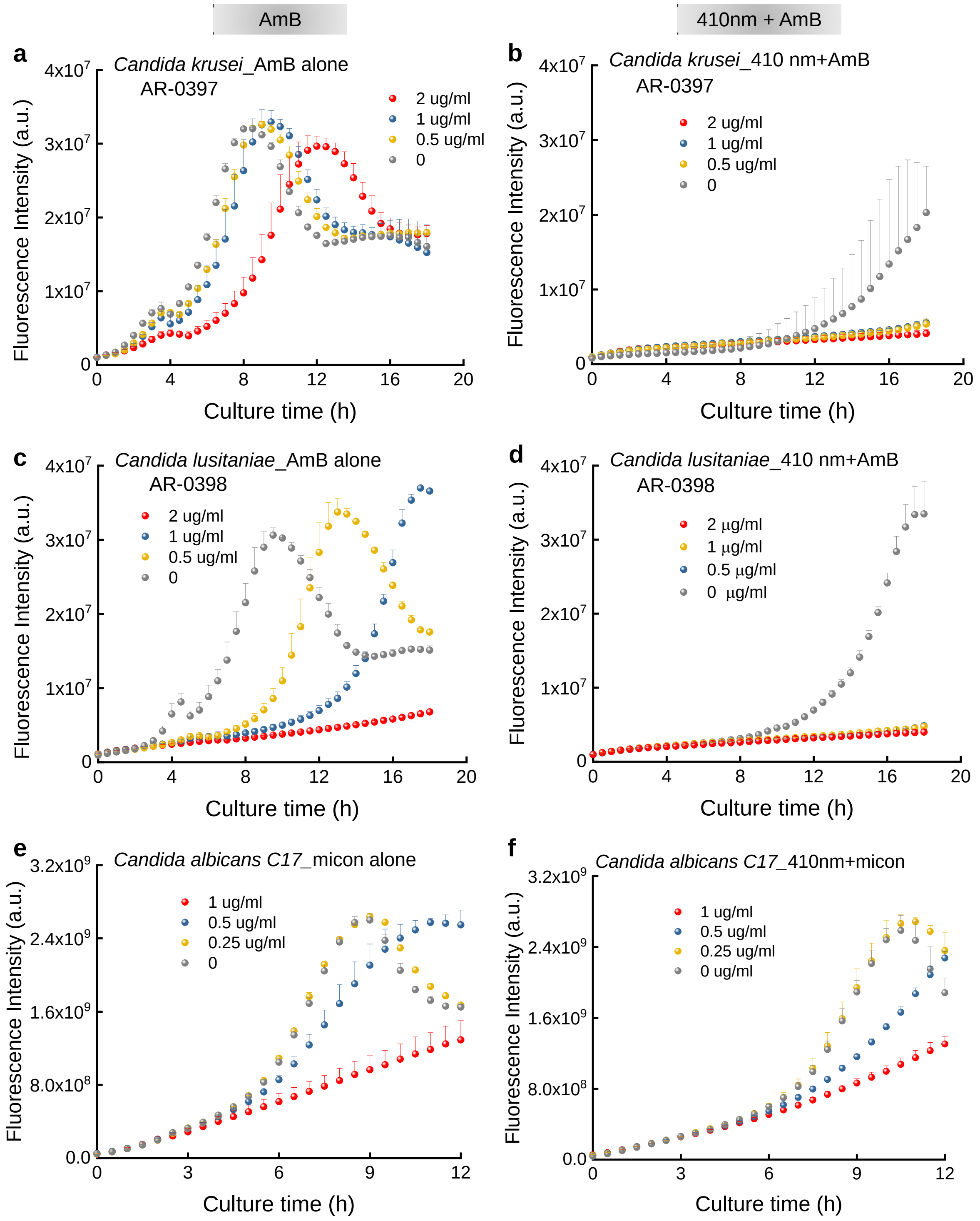

Supplementary Figure 4. Time-course PrestoBlue fluorescence intensity from various fungal species under the treatment of 410 nm and antifungal drugs. Abbreviations: AmB (amphotericin B), micon (miconazole).

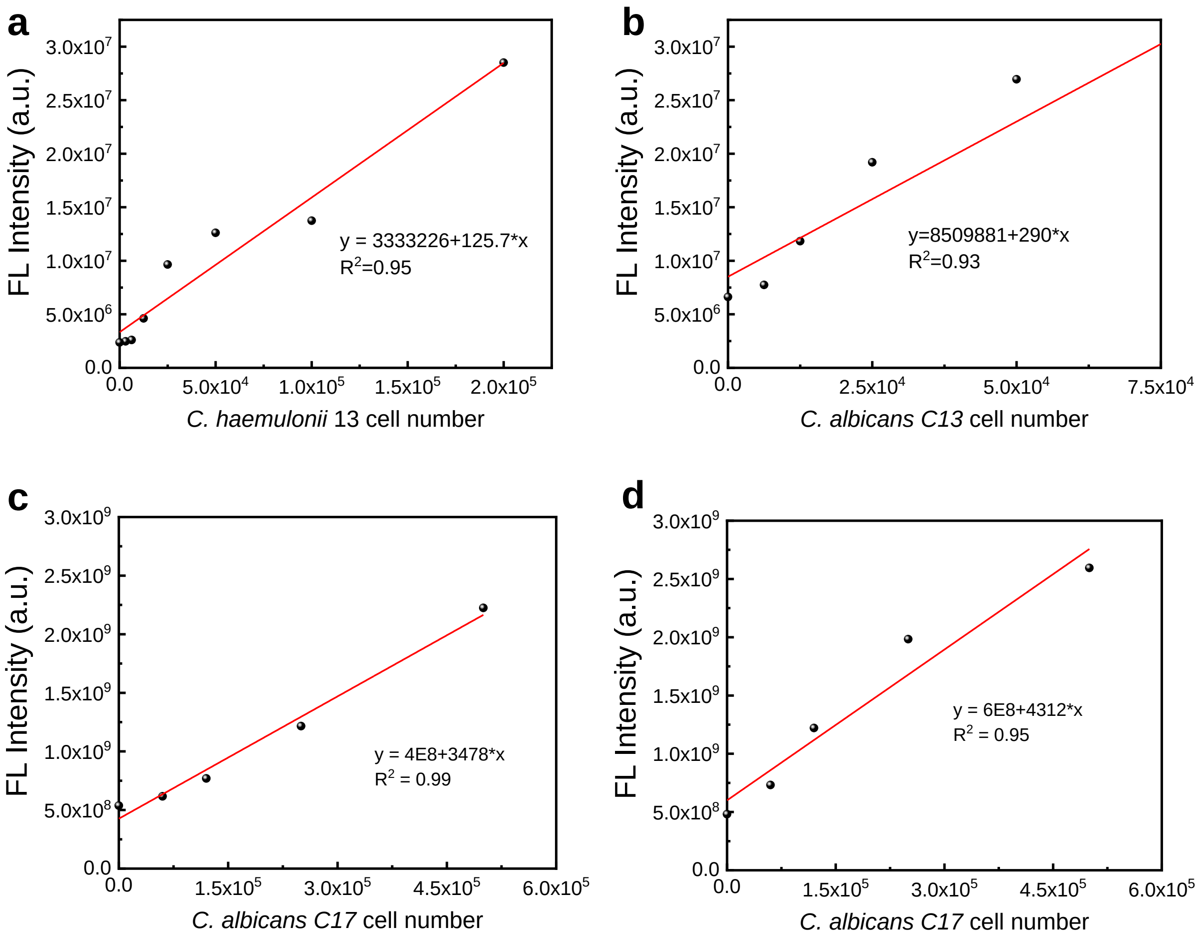

Supplementary Figure 5. Calibration curves of *Candida spp.* CFU/ml versus PrestoBlue fluorescence intensity.

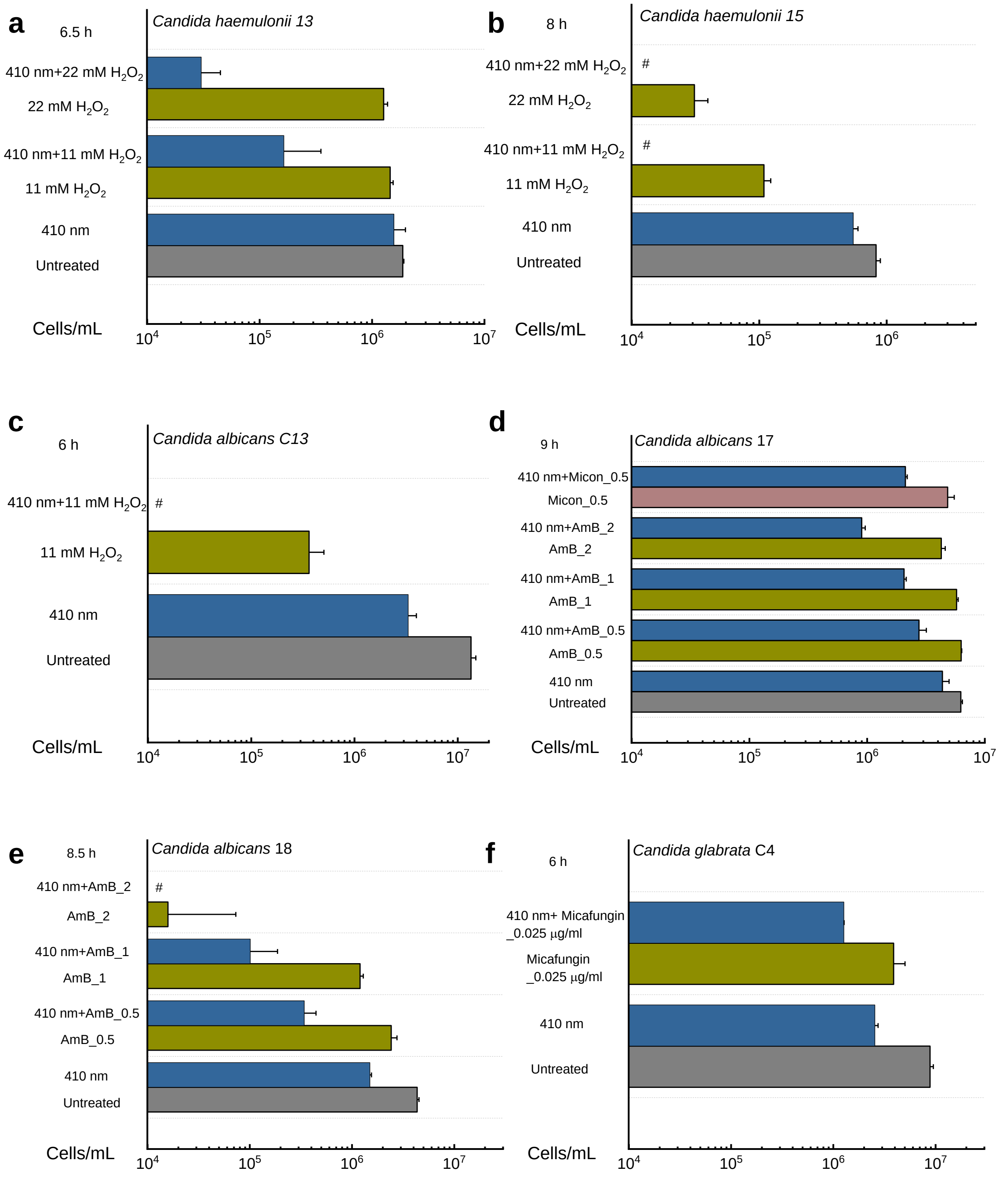

Supplementary Figure 6. Derived *Candida* cells/mL after different treatments based on the calibration curves. Data: Mean+SD. N=3. Pound sign (#) means below the detection limit.

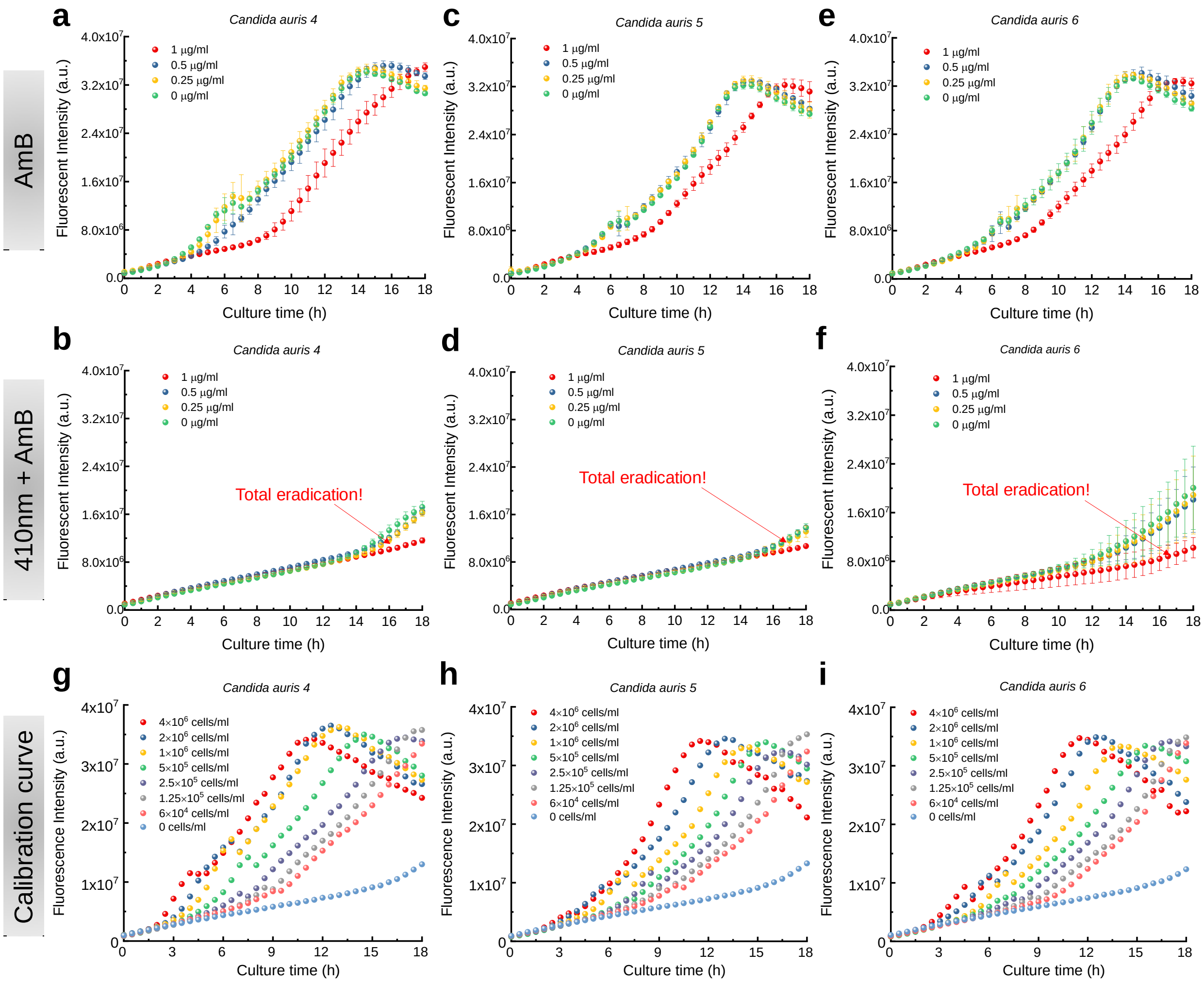

Supplementary Figure 7. Time-course PrestoBlue fluorescence intensity from various *Candida auris* strains under the treatment of 410 nm and antifungal drugs. Abbreviation: AmB (amphotericin B).

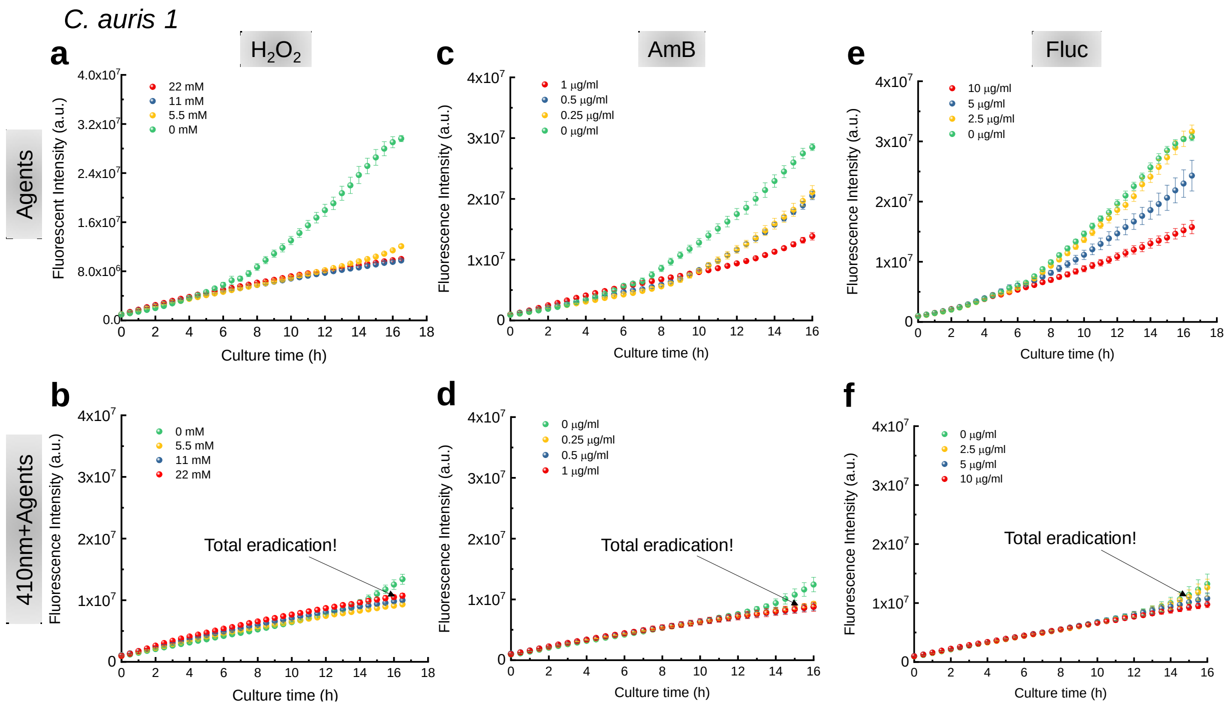

Supplementary Figure 8. Time-course PrestoBlue fluorescence intensity from *Candida auris* 1 under the treatment of 410 nm and antifungal agents. Abbreviations: AmB (amphotericin B), Fluc (fluconazole).

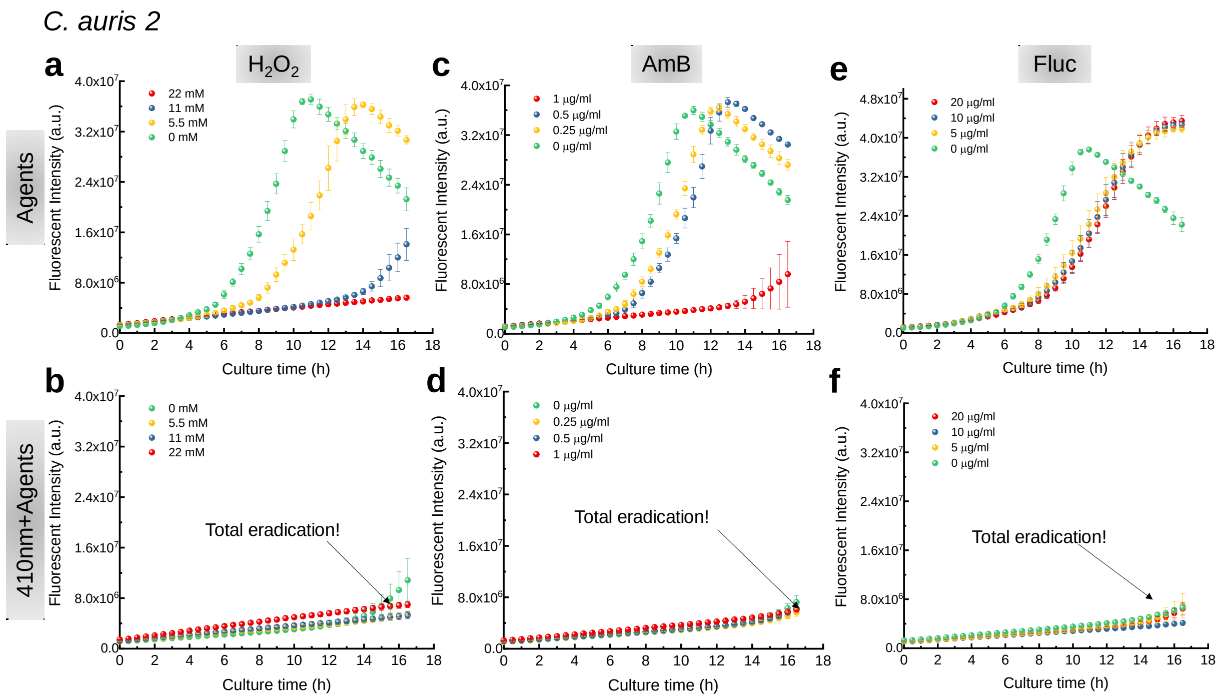

Supplementary Figure 9. Time-course PrestoBlue fluorescence intensity from *Candida auris 2* under the treatment of 410 nm and antifungal agents. Abbreviations: AmB (amphotericin B), Fluc (fluconazole).

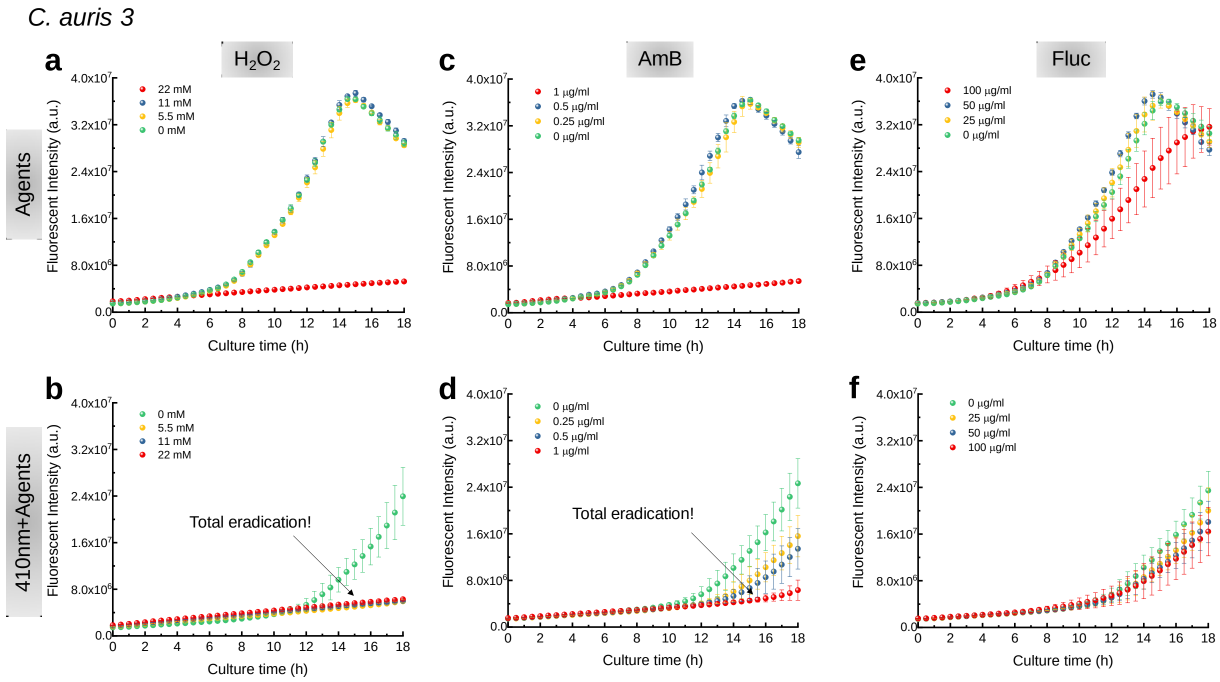

Supplementary Figure 10. Time-course PrestoBlue fluorescence intensity from *Candida auris* 3 under the treatment of 410 nm and antifungal agents. Abbreviations: AmB (amphotericin B), Fluc (fluconazole).

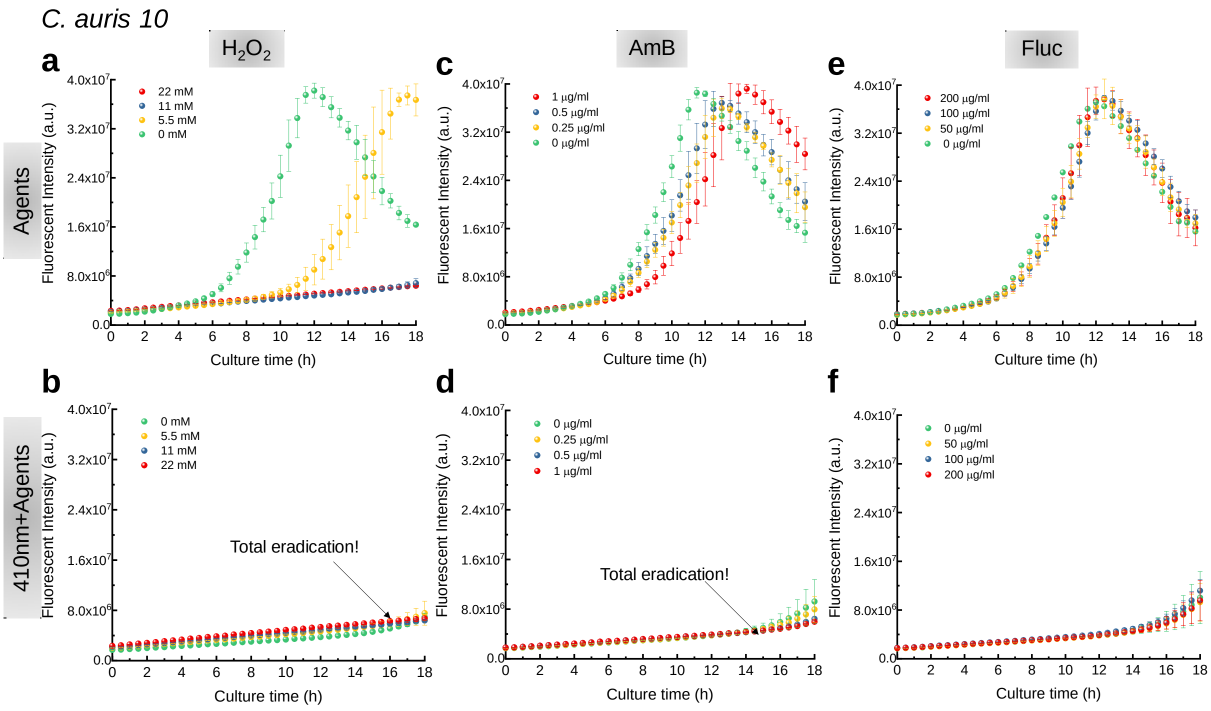

Supplementary Figure 11. Time-course PrestoBlue fluorescence intensity from *Candida auris* 10 under the treatment of 410 nm and antifungal agents. Abbreviations: AmB (amphotericin B), Fluc (fluconazole).

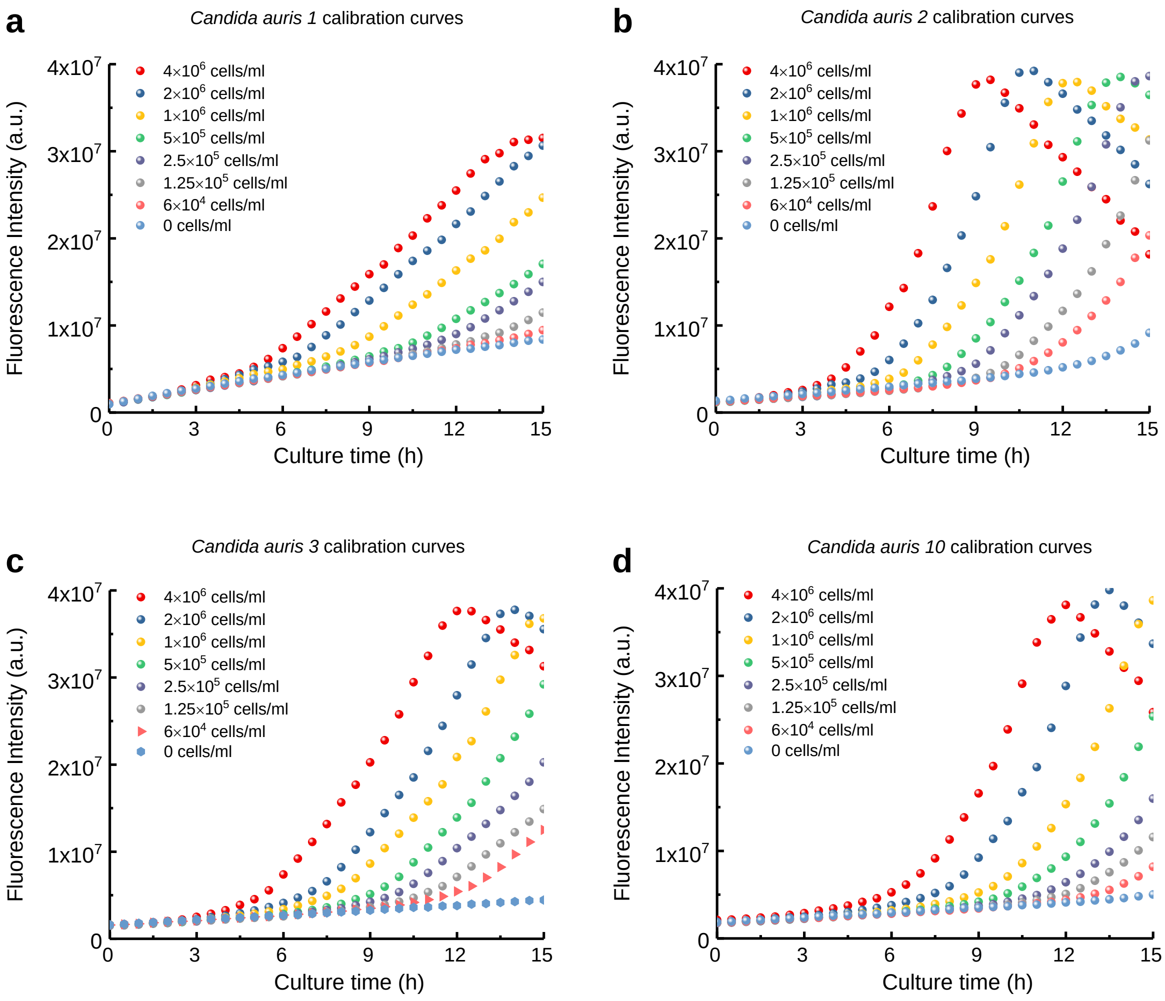

Supplementary Figure 12. Time-course PrestoBlue fluorescence intensity of *Candida auris* strains at specific concentrations.

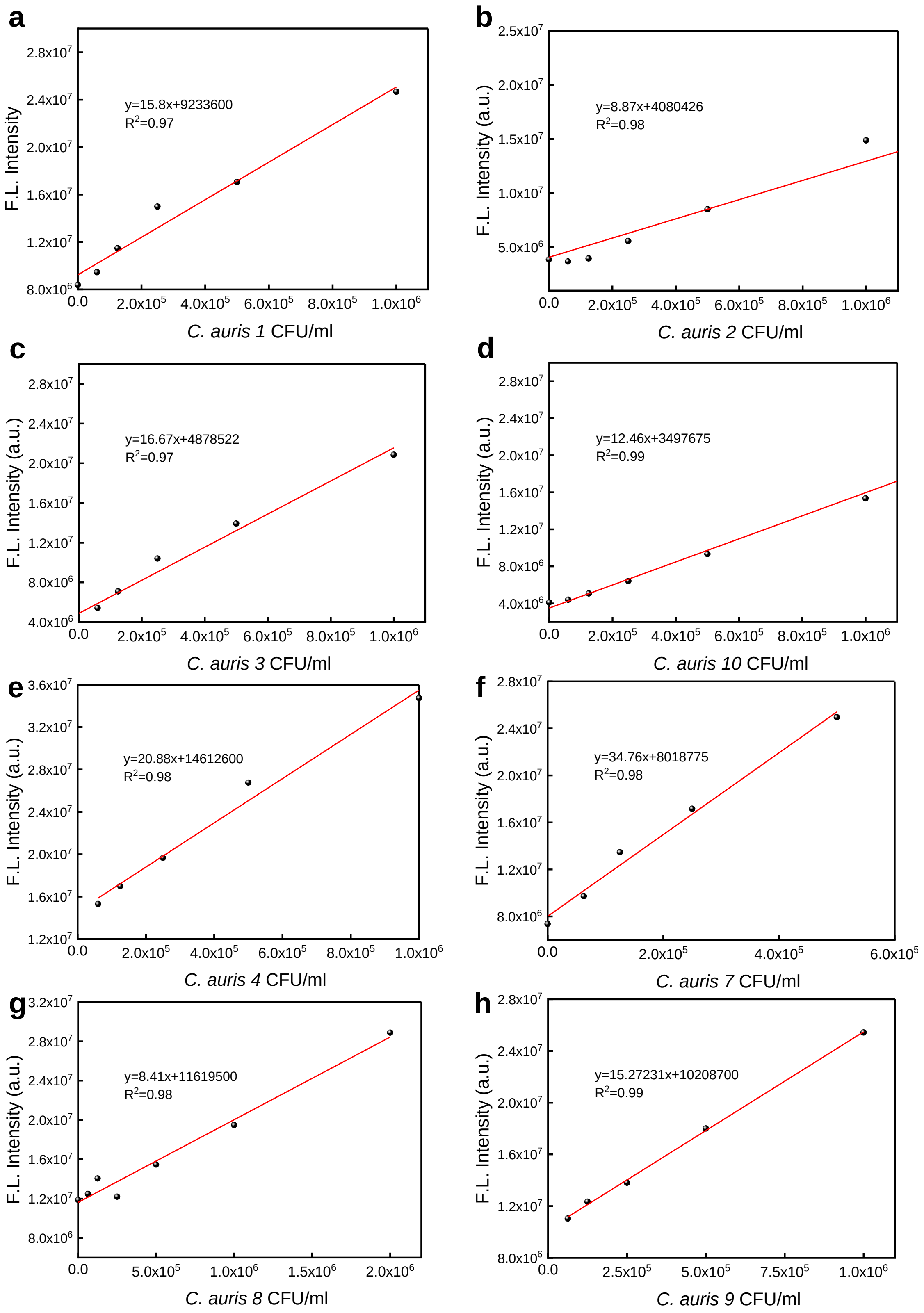

Supplementary Figure 13. Calibration curves of *C. auris* CFU/ml versus prestoblue fluorescence intensity.
